# Lipocalin-2 promotes acute lung inflammation and oxidative stress by enhancing macrophage iron accumulation

**DOI:** 10.7150/ijbs.79915

**Published:** 2023-02-13

**Authors:** Hyeong Seok An, Jung-Wan Yoo, Jong Hwan Jeong, Manbong Heo, Si Hwan Hwang, Hye Min Jang, Eun Ae Jeong, Jaewoong Lee, Hyun Joo Shin, Kyung Eun Kim, Meong Cheol Shin, Gu Seob Roh

**Affiliations:** 1 Department of Anatomy & Convergence Medical Science, College of Medicine, Institute of Health Sciences, Gyeongsang National University, Jinju, 52727, Republic of Korea; 2 Division of Pulmonary and Critical Care Medicine, Department of Internal Medicine, College of Medicine, Gyeongsang National University Hospital, Gyeongsang National University, Jinju, 52727, Republic of Korea; 3 Department of Medicine, College of Medicine, Gyeongsang National University, Jinju, 52727, Republic of Korea; 4 College of Pharmacy, Research Institute of Pharmaceutical Sciences, Gyeongsang National University, Jinju, 52828, Republic of Korea

**Keywords:** Lipocalin-2, Iron, Macrophage, Inflammation, Oxidative stress, Acute lung injury

## Abstract

Lipocalin-2 (LCN2) is an acute-phase protein that regulates inflammatory responses to bacteria or lipopolysaccharide (LPS). Although the bacteriostatic role of LCN2 is well studied, the function of LCN2 in acute lung damage remains unclear. Here, LCN2 knockout (KO) mice were used to investigate the role of LCN2 in LPS-treated mice with or without recombinant LCN2 (rLCN2). In addition, we employed patients with pneumonia. RAW264.7 cells were given LCN2 inhibition or rLCN2 with or without iron chelator deferiprone. LCN2 KO mice had a higher survival rate than wild-type (WT) mice after LPS treatment. In addition to elevated LCN2 levels in serum and bronchoalveolar lavage fluid (BALF), LPS treatment also increased LCN2 protein in alveolar macrophage lysates of BALF. LCN2 deletion attenuated neutrophil and macrophage infiltration in the lungs of LPS-treated mice as well as serum and BALF interleukin-6 (IL-6). Circulating proinflammatory cytokines and LCN2-positive macrophages were prominently increased in the BALF of pneumonia patients. In addition to increase of iron-stained macrophages in pneumonia patients, increased iron-stained macrophages and oxidative stress in LPS-treated mice were inhibited by LCN2 deletion. In contrast, rLCN2 pretreatment aggravated lung inflammation and oxidative stress in LPS-treated WT mice and then resulted in higher mortality. In RAW264.7 cells, exogenous LCN2 treatment also increased inflammation and oxidative stress, whereas LCN2 knockdown markedly diminished these effects. Furthermore, deferiprone inhibited inflammation, oxidative stress, and phagocytosis in RAW264.7 cells with high LCN2 levels, as well as LPS-induced acute lung injury in WT and LCN2 KO mice. Thus, these findings suggest that LCN2 plays a key role in inflammation and oxidative stress following acute lung injury and that LCN2 is a potential therapeutic target for pneumonia or acute lung injury.

## Introduction

Acute lung injury usually causes acute respiretory distress syndrome (ARDS), which requires intensive care and has a high mortality 1, 2. Acute lung injury is characterized by neutrophil infiltration, epithelial damage, and increased alveolar permeability 3. Lung resident macrophages communicate with the immune microenvironment to suppress or exacerbate inflammatory responses. Alveolar macrophages and monocyte-derived macrophages polarize into classically lipopolysaccharide (LPS)-induced activated macrophage M1 phenotype or into alternatively M2 phenotype in the presence of interleukin (IL)-4 and IL-13 4, 5. In particular, the elevation of M1-like genes using genome-wide transcriptional profiling is associated with death on the first day in alveolar macrophages of the BALF taken from patients with ARDS 6, 7. Although activated macrophages play a role in inflammation and phagocytosis in response to acute lung damage, the molecular mechanisms responsible for engaging activated macrophages are still poorly understood.

Lipocalin-2 (LCN2) is a secreted acute-phase protein also referred to as neutrophil gelatinase-associated lipocalin 8. LCN2 functions in multiple biological processes, including transport of iron, immune defense against bacterial infections, and cell migration and differentiation 9. However, LCN2 plays both protective and detrimental roles in the inflammatory response. For example, LCN2 limits iron uptake in the host, which reduces bacterial growth during the innate immune response 10-12. In experimental models of pneumonia or sepsis, LCN2 deficiency increases bacterial proliferation and lowers host survival 13-17. In contrast, LCN2 deletion has been shown to markedly attenuate the production of proinflammatory cytokines in mice with ARDS 18. Based on these inconsistent evidences, the precise role of LCN2 in acute lung injury is not fully understood.

Iron accumulation is associated with several inflammatory respiratory diseases, including ARDS, and mediates an inflammatory response that produces reactive oxygen species through the Fenton reaction 19, 20. The iron-overloaded lung is vulnerable to oxidative stress since it is constantly exposed to the highest concentration of oxygen. Although the lung expresses high levels of several antioxidants, such as ascorbate, heme oxygenase-1 (HO-1), superoxide dismutase (SOD), and iron-binding proteins, an imbalance of iron and antioxidants leads to oxidative stress 21. Although antioxidants have been suggested as therapeutics for acute lung injury, to our knowledge no studies have been evaluated to yet examining at the efficacy of iron chelators in acute lung injury.

In this study, we investigated the role of LCN2 and its underlying mechanism of action in LPS-treated LCN2-deficient mice with recombinant LCN2 (rLCN2) as well as pneumonia patients. In particular, we found that rLCN2 pretreatment promotes inflammation and oxidative stress in LPS-treated RAW264.7 cells, but iron chelator deferiprone decreased inflammation and phagocytic function. Based on these findings, we hypothesize that LCN2 deletion could protect against pneumonia by inhibiting inflammation and oxidative stress.

## Materials and Methods

### Human patients

From June to November 2020, we obtained blood and bronchoalveolar lavage fluid (BALF) samples from 26 patients with severe pneumonia who were admitted to the medical ICU due to acute respiratory failure. Patients with pneumonia received high-flow nasal cannula oxygen or invasive mechanical ventilation to treat their respiratory failure (Supplementary Table 1). Bronchoscopy with BALF collection was performed to evaluate bronchial lesions, obtain respiratory samples, and remove secretions. As controls, ten patients admitted to the general ward during the same period were selected. These control patients underwent bronchoscopy with BALF collection to evaluate stable chronic lung disease. Demographic data (age, sex, body mass index, comorbidities) and clinical characteristics (septic shock, ARDS, types of oxygen therapy) were recorded (Supplementary Table 1). Various laboratory parameters (white cell count, hemoglobin, platelet, C-reactive protein, albumin, and procalcitonin) were collected and analyzed retrospectively (Supplementary Table 1). The human study protocol was approved by the institutional review board of Gyeongsang National University Hospital (GNUH) (2020-11-009), and written informed consent was waived due to the retrospective nature of the analysis of clinical data and the use of blood and BALF samples provided by the Biobank of GNUH.

### Experimental mouse model

Female and male LCN2 KO mice were purchased from The Jackson Laboratory (Bar Harbor, ME, USA). LCN2 (+/+) and LCN2 (-/-) mice on a C57BL/6J background were back-crossed to produce homozygous and heterozygous mice free of background effects for 8 to 10 generations. The absence of LCN2 was confirmed by PCR analysis of genomic DNA. According to the National Institutes of Health Guidelines on the Use of Laboratory Animals, the animal research was carried out at Gyeongsang National University (GNU). The animal study protocol (GNU-190701-M0033) was authorized by the Animal Care Committee of the GNU. After genotyping at 5 weeks of age, male LCN2 KO and WT mice were randomly divided into four groups (n = 6-8). Six-month-old mice were intratracheally administered LPS (1 mg/kg, Sigma-Aldrich, St. Louis, MO, USA) or 0.9% normal saline as control. After 24 hours, mice were sacrificed, and samples, including serum, BALF, and lung tissues, were obtained. To determine the percentage of survival, LPS (20 mg/kg) was intratracheally administered to WT and LCN2 KO mice (n = 10), and these mice were monitored for 8 days. In another study, recombinant LCN2 (rLCN2, 300 μg/kg) was intraperitoneally injected into WT and LCN2 KO mice (n = 6-8). After 24 hours, saline or LPS (1 mg/kg) was administered, and the mice were sacrificed 24 hours later. To investigate the mortality rate after rLCN2 pretreatment, LPS (20 mg/kg) was administrated to WT and LCN2 KO mice (n = 10), which were monitored for 8 days. In order to evaluate the protective effect of an iron chelator against acute lung injury, LPS (1 mg/kg) was intratracheally administered to WT and LCN2 KO mice (n = 3-4) and then iron chelator deferiprone (100 mg/kg, DFP, Sigma-Aldrich) was intraperitoneally injected after 3 hours of LPS instillation. Mice were sacrificed at 24 hours and only BALF was obtained.

### Recombinant LCN2 production

The pET28a-SUMO-LCN2-ABD vector was prepared by insertion of the mouse *lcn2* gene (545 bp, GenScript, Piscataway, NJ, USA) into a linearized pET28a-SUMO-ABD vector. The vector was transformed to a BL21 (DE3) *E. coli* strain for the protein expression. For every production, a starter culture was prepared by inoculating 50 mL of Lura-Bertani (LB) medium (80 µg/mL kanamycin) with a colony of the transformed *E coli*. After incubation of the culture at 37ºC overnight, the starter culture was added to 1 L of fresh LB medium (80 µg/mL of kanamycin). The culture was maintained at the same incubation conditions, and, when the UV absorbance at 600 nm reached about 1, isopropylthio-D-galactoside (inducer) was added (final concentration: 0.5 mM). After further incubation for 4 h, the *E. coli* cells were pelleted by centrifugation (4000 rpm for 20 min). The cells were then resuspended in phosphate buffer saline (20 mM phosphate, 300 mM NaCl, 1% soybean protease inhibitor, 1% leupeptin, pH 7), and lysed by sonication (4 cycles with 30 sec run at 50% output on ice). The lysed sample was centrifuged (8000 rpm for 10 min) and the supernatant was loaded to the Talon^®^ metal affinity resins (Clontech, Mountain View, CA, USA) for purification. The expression and purification of the SUMO-LCN2-ABD (recombinant LCN2, rLCN2) was verified by SDS-PAGE.

### Sample preparation

All mice were anesthetized with Zoletil (20 mg/kg, Virbac Laboratories, Carros, France) and Rompun (5 mg/kg, Bayer, Bayer Korea, Republic of Korea). Blood samples were extracted from the left ventricle and centrifuged. For protein extraction, the right lung was isolated, frozen in liquid nitrogen, and stored at -80℃. For histological analysis, the left lung was slowly perfused with 4% paraformaldehyde and immersed in a fixative solution overnight. Lung tissues were embedded in paraffin and cut into 5-µm sections.

### BALF analysis

After the removal of blood, the BALF was collected by instillation and suction of 0.9% normal saline (3 × 0.8 mL) in the lungs with 24-G intravenous catheter. BALF samples were centrifuged for 5 minutes at 400 xg at 4°C, and supernatants were collected and stored at -80℃ until use. The remaining cell pellets were used to prepare cytospin slides to identify cells present in the pulmonary air spaces. Using cytospin (Shandon Cytospin 3 Cyto-centrifuge, Thermo Fisher Scientific, Waltham, MA, USA), slides were prepared by centrifuging pellets at 1,165 xg for 10 minutes. After air-drying, cytospin slides were stained using H&E (Abcam, Cambridge, MA, USA) and Differential Quik stain (Diff-Quik, Polysciences, Inc., Warrington, PA, USA). Cytospin slides were visualized under a BX51 light microscope (Olympus, Tokyo, Japan).

### Isolation of mature macrophages in the BALF

Cells from the BALF were cultured in RPMI 1640 medium (RPMI; Gibco, Life Technologies, MD, USA) supplemented with 10% fetal bovine serum (Gibco), 1% penicillin/streptomycin (Gibco), L-Glutamine 200 mM (Gibco). Mature macrophages were encouraged to adhere to cell culture plates for 2 hours at 37 °C, at which time the media was discarded, and the cells underwent two RPMI medium washes. Collected macrophages were lysed in lysis buffer for western blot analysis.

### Fluorescence-Activated Cell Sorting (FACS) analysis

The cells collected from the BALF were used for flow cytometry. The fluorescent antibodies were purchased from BioLegend (San Diego, CA, USA); Alexa Fluor® 647 anti-mouse CD170 (Siglec-F), 155519; Brilliant Violet 785™ anti-mouse CD11c; 117335; PE/Cyanine5 anti-mouse CD86, 105015; Brilliant Violet 711™ anti-mouse CD206 (MMR), 141727. Dead/Live staining (eBioscience™ Fixable Viability Dye eFluor™ 780) from Invitrogen (Carlsbad, CA, USA) was used to separate dead and live cells. The fluorescence wavelength of the stained cells was measured by BD FACSymphony™ A3 Cell Analyzer (San jose, CA, USA). The FlowJo_v10.8.1 were utilized to set the gates.

### Enzyme-Linked Immunosorbent Assay (ELISA) analysis

Serum and BALF cytokines were measured using mouse IL-6 (ab100713, Abcam), IL-1β (ab197742, Abcam), IL-10 (ab100697, Abcam), LCN2 (R&D Systems, Minneapolis, MN, USA), human IL-6 (ab46042, Abcam), IL-1β (ab214025, Abcam), TNF-α (ab181421, Abcam), IL-10 (ab46034, Abcam), and LCN2 (R&D Systems) ELISA kits according to the manufacturers' protocols.

### Western blot analysis

Frozen lung tissues or cells were homogenized in T-PER (Tissue Protein Extraction Reagent, Pierce, Rockford, IL, USA). Homogenized samples were centrifuged for 30 minutes at 13,475 xg at 4°C. Lysates were probed with primary antibodies (Supplementary Table 2). Using an enhanced chemiluminescence substrate, membranes were seen (Pierce). All band densitometry were measured using the Multi-Gauge image analysis program (Fujifilm, Tokyo, Japan, v3.0). β-actin was used as an internal control to normalize protein levels.

### Immunohistochemistry

Deparaffinized lung sections and BALF cytospin slides were placed in a 0.3% H_2_O_2_ solution for 10 minutes. After washing, slides were treated with primary antibodies (Supplementary Table 2) diluted in blocking serum for overnight at 4°C in a humidified chamber. After incubation with a secondary biotinylated antibody (1:200), section slides were incubated in avidin-biotin-peroxidase complex solution (ABC solution, Vector Laboratories, Burlingame, CA, USA). Slides were developed with 3,3-diaminobenzidine (DAB) peroxidase substrate kit (Vector Laboratories), dehydrated in varying alcohol concentrations, cleared in xylene, and cover-slipped with Permount mounting (Sigma-Aldrich). The immunostaining sections were visualized under BX51 light microscopy (Olympus).

### Immunofluorescence

Deparaffinized sections and BALF cytospin slides were incubated overnight at 4°C with primary antibodies (Supplementary Table 2). After washing, slides were incubated with Alexa Fluor donkey anti-secondary antibody (1:1000, Invitrogen). 4′, 6-diamidino-2-phenylindole (DAPI, 1:10,000; Invitrogen) was used to stain the nuclei. Slides were examined under under a BX51-DSU microscope (Olympus) and images were captured.

### Iron staining and quantification

Deparaffinized lung sections and BALF cytospin slides were stained with Perls Prussian blue (Iron Stain Kit, Abcam). The iron stained slides were visualized under a BX51 light microscope (Olympus). Total iron levels from frozen lungs were measured using an iron assay kit (MAK025, Sigma-Aldrich) according to the manufacturer's instructions.

### Cell culture

The macrophage cell line, RAW264.7 cells, was cultured in Dulbecco's modified Eagle medium (DMEM; Gibco) supplemented with 1% penicillin/streptomycin (Gibco) and 10% fetal bovine serum (Gibco) at 37 ℃ in a 5% CO_2_ humidified incubator. RAW264.7 cells were plated at a density of 0.5x10^6^ cells per 60-mm dish. RAW264.7 cells were stimulated with LPS (100 ng/mL, Sigma-Aldrich) at the specified time points to secrete LCN2. Following LPS treatment for 24 hours, their medium containing LCN2 (LTM) was incubated with RAW264.7 cells for the indicated times.

### Transfection of small interfering RNA

Small interfering RNA (siRNA) targeting mouse LCN2 (Santa Cruz Biotech., Santa Cruz, CA, USA) were purchased. RAW264.7 cells were transfected with LCN2 siRNA or control scrambled siRNA using lipofectamine RNAiMAX (Invitrogen) according to the manufacturer's instructions.

### Treatment of recombinant LCN2 (rLCN2) and iron chelator deferiprone (DFP)

rLCN2 was incubated with 0.5, 1, 2, or 4 μg/mL mouse rLCN2 for 24 hours. RAW264.7 cells treated with DFP (Sigma-Aldrich) were pre-treated with 50 µM DFP for 1 hour before treatment with LTM or 2 µg/mL mouse LCN2 for 12 hours.

### Mitochondrial superoxide (MitoSOX) and Phagocytosis assay

For measurement of LCN2 mediated-mitochondrial superoxide, RAW264.7 cells were treated with 5 μM MitoSOX (Invitrogen) at 37 °C for 30 min. To determine phagocytic function of DFP or siLCN2, RAW 264.7 cells were incubated with 20 μg/mL Zymosan (Invitrogen) at 37 °C for 30 min. Nuclei were stained with DAPI (1:10,000, Invitrogen) following fixation with 4% paraformaldehyde for 10 min. Fluorescent slides were visualized using a BX51-DSU microscope (Olympus).

### Statistical analysis

For statistical analyses, we used PRISM 7.0 (GraphPad Software Inc., San Diego, CA, USA). Group differences were determined using unpaired Student *t*-tests and two-way ANOVA followed by Tukey's *post-hoc* tests. All results were presented as mean ± standard error of the mean (SEM). A *P*-value of less than 0.05 was used to indicate statistical significance.

## Results

### LCN2 deletion attenuates acute lung inflammation in LPS-treated mice

**Figure 1:**
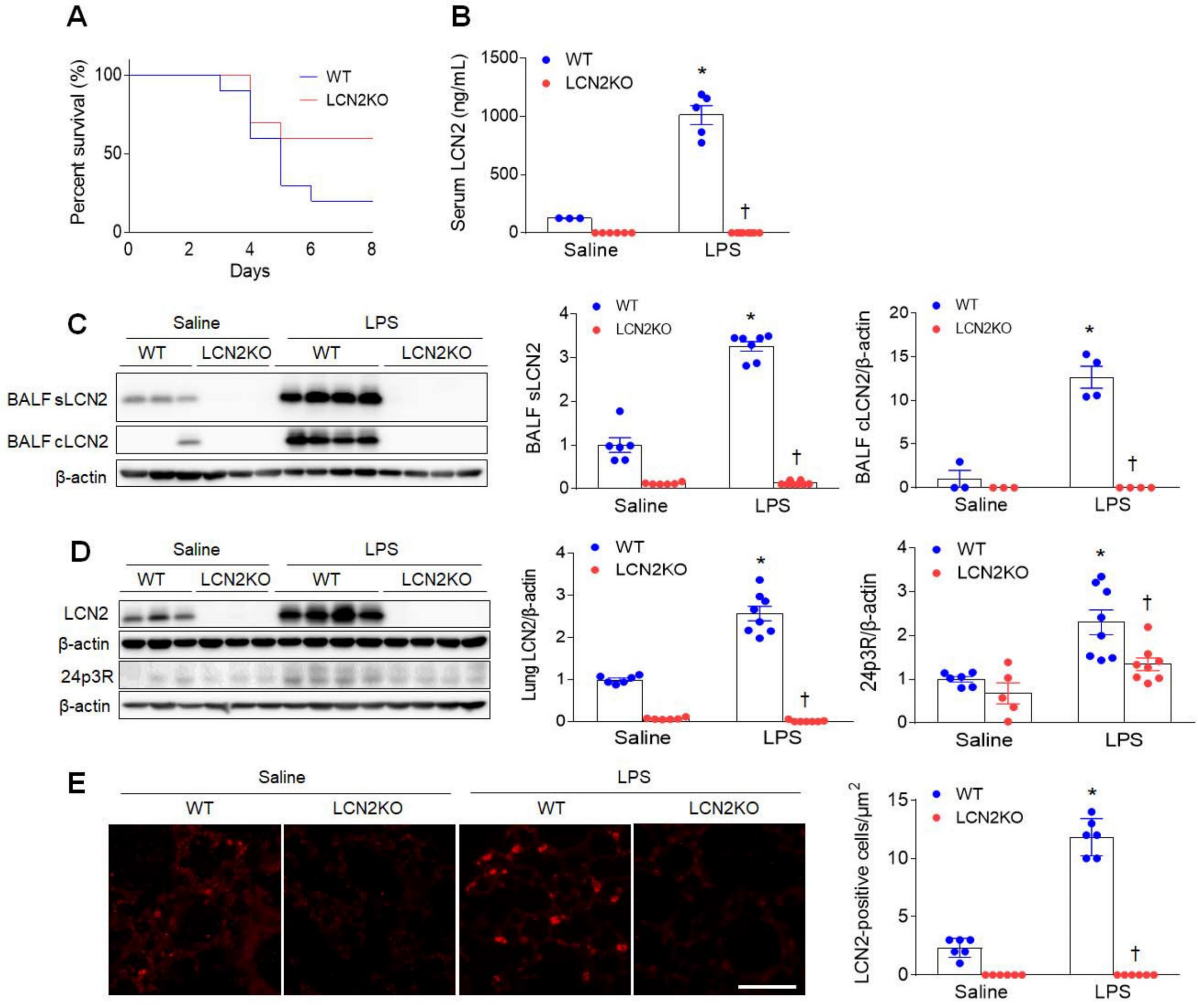
** LCN2 increases in the BALF and lung of LPS-treated mice. (A)** Percent survival in LPS-treated WT and LCN2 KO mice for 8days. Mice (n = 10) were intratracheally given with 20 mg/kg LPS. **(B)** ELISA analysis of serum LCN2 protein levels (n = 3-8). **(C)** Western blot and quantification of supernatant (s) and alveolar macrophage lysates (c) LCN2 proteins in the BALF (n = 3-8). β-actin served as a loading control. **(D)** Western blot and quantification of LCN2 and its receptor 24p3R proteins in lung tissues (n = 5-8). β-actin served as a loading control. **(E)** Representative images of LCN2 immunofluorescence. Scale bar, 50 μm. LCN2-positive cells were counted and analyzed in three fields (100x100 μm^2^) for each slide (n = 6). Differences between four groups were evaluated using two-way ANOVA followed by Tukey's multiple comparisons test. *P < 0.05 versus WT saline. †*P* < 0.05 versus WT LPS. All data are presented as mean ± SEM.

To first determine the effect of LCN2 deletion on survival, mice were given an intratracheal injection of LPS (20 mg/kg). Eight days after LPS injection, LCN2 KO mice had a higher percent survival than LPS-treated WT mice (Figure 1A). Next, to evaluate whether LCN2 deletion affects acute lung inflammation, mice were treated with intratracheal LPS (1 mg/kg) injection. As well as increased serum LCN2 levels (Figure 1B), LCN2 proteins from supernatant and cell lysates of the BALF were significantly increased in LPS-treated WT mice compared to saline-treated WT mice (Figure 1C). As expected, lung LCN2 and its receptor 24p3R protein expressions were also elevated in LPS-treated WT mice (Figure 1D). Immunofluorescence study showed that LCN2-positive cells were significantly increased in LPS-treated WT mice (Figure 1E). Furthermore, Diff-Quik staining and immunofluorescence revealed that many Ly6G-positive neutrophils and F4/80-positive macrophages in the BALF of LPS-treated WT mice were attenuated by LCN2 deletion (Supplementary Figure 1). Using ELISA analysis, LCN2 deletion significantly inhibited the upregulation of serum and BALF IL-6 levels in LPS-treated mice (Supplementary Figure 2). To further determine whether LCN2 deletion affects the polarization of alveolar macrophages in the BALF, we performed FACS (Supplementary Figure 3). BAL cells were analyzed for Siglec F^+^ CD11c^+^ (alveolar macrophages), Siglec F^+^ CD86^+^ (M1 polarization), and Siglec F^+^ CD206^+^ (M2 polarization) (Supplementary Figure 3A). Similar to human BALF (Supplementary Table 1), the proportion of macrophages tended to be decreased in LPS-treated WT mice compared to saline-treated mice. However, the proportion of alveolar macrophage in LCN2KO mice was not changed by LPS. In particular, there was no difference between the M1 macrophage and M2 macrophage portions due to the small population of alveolar macrophages in BALF (Supplementary Figure 3B-D). Taken together, these findings indicate that LCN2-positive neutrophils and alveolar macrophages play an important role in the resolution of acute lung inflammation.

### Circulating LCN2 level is elevated in patients with pneumonia

These biologically significant findings in LPS-induced acute lung injury were validated in human patients with pneumonia. Notably, pneumonia patients had significantly higher serum LCN2 levels than control subjects (Supplementary Table 1). In addition to H&E staining, many LCN2-positive cells were significantly increased in BALF slides from pneumonia patients (Supplementary Figure 4A). As shown in supplementary table 1, pneumonia patients had considerably greater white blood cell counts, C-reactive protein levels, and procalcitonin levels than control subjects. A high rate of (65.4%, 17/26) of pneumonia patients progressed to ARDS due to severe inflammation. In particular, differential cell analysis in the BALF revealed a significantly higher proportion of neutrophils in patients with pneumonia compared to control subjects (Supplementary Table 1). Consistent with LPS-treated mice, serum and BALF IL-1β and IL-6 levels in pneumonia patients were higher than in controls (Supplementary Figure 4B, C). In particular, there was a significant increase of tumor necrosis factor (TNF)-α in the BALF of pneumonia patients. These results suggest that LCN2 may play a role in the regulation of acute lung inflammation in pneumonia patients.

### LCN2 deletion attenuates oxidative stress and iron accumulation in the lung of LPS-treated mice

**Figure 2:**
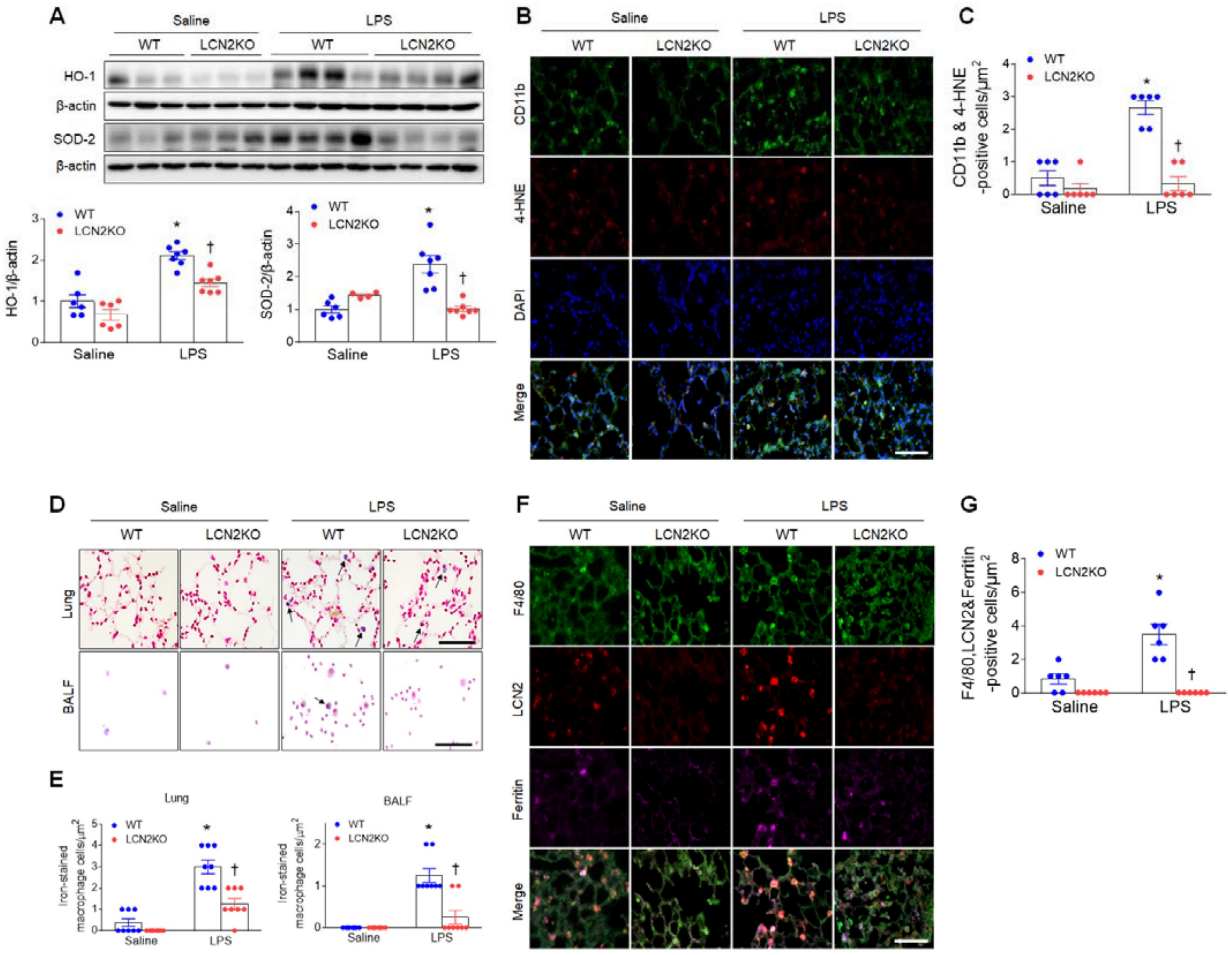
** LCN2 deletion attenuates lung oxidative stress and iron accumulation in LPS-treated mice. (A)** Western blot and quantification of HO-1 and SOD-2 protein levels. β-actin served as a loading control. (n = 4-7). **(B)** Representative double immunofluorescence images of CD11b and 4-HNE in lung sections. Nuclei were stained with DAPI. Scale bar, 50 μm. **(C)** Co-localized CD11b and 4-HNE-positive cells were counted and analyzed in three fields (100x100 μm^2^) for each slide (n = 6). **(D)** Representative images of Perls Prussian blue staining in lung sections and BALF slides. Arrows indicate iron-stained alveolar macrophages. Scale bar, 50 μm. **(E)** In lung tissues and BALF slides (D), iron-stained macrophages were counted and analyzed in four fields (100x100 μm^2^) for each slide (n = 6). **(F)** Representative triple immunofluorescence images of F4/80, LCN2, and ferritin in lung sections. Scale bar, 50 μm. **(G)** Co-localized F4/80, LCN2, and ferritin-positive cells were counted and analyzed in three fields (100x100 μm^2^) for each slide (n = 6). Differences between four groups were evaluated using two-way ANOVA followed by Tukey's multiple comparisons test. *P < 0.05 versus WT saline. †*P* < 0.05 versus WT LPS. All data are presented as mean ± SEM.

LPS treatment produces proinflammatory cytokines and recruits neutrophils to the alveolar spaces to initiate inflammatory responses 22. In particular, persistent neutrophil accumulation produces reactive oxygen radicals and activates alveolar macrophages 23. Increased protein levels of heme oxygenase-1 (HO-1) and superoxide dismutase-2 (SOD-2) in the lung of LPS-treated WT mice were significantly reduced by LCN2 deletion (Figure 2A). In addition, many 4-hydroxynonenal (4-HNE)-positive macrophages (CD11b-positive cells) in LPS-treated WT mice were also significantly reduced by LCN2 deletion (Figure 2B, C). Similarly, in human BALF, immunofluorescence showed that HO-1- or 4-HNE-positive cells were increased in pneumonia patients compared to control subjects (Supplementary Figure 5A, B).

LCN2 not only transports secreted iron but also regulates intracellular iron concentrations 24. Inflammation and oxidative stress were induced by iron accumulation 25. So, to determine whether LCN2 affects iron overload in LPS-induced acute lung injury, we performed Perls Prussian blue iron staining (Figure 2D). Notably, we found that the induction of iron-stained alveolar macrophages in the lung section and BALF of LPS-treated WT mice was significantly reduced by LCN2 deletion (Figure 2E). Many iron-stained macrophages were also observed in the BALF of pneumonia patients (Supplementary Figure 5C).

To further determine whether LCN2-positive cells affect iron uptake in alveolar macrophages of LPS-treated mice, we conducted triple immunofluorescence with F4/80, LCN2, and ferritin (iron storage protein) antibodies. Many F4/80 and LCN2-positive macrophages were co-localized with ferritin-positive cells in LPS-treated WT mice. However, these co-localized macrophages were significantly reduced by LCN2 deletion (Figure 2F, G). Taken together, these findings suggest that LCN2 may play a critical role in the regulation of iron-mediated oxidative stress in acute lung injury.

### Recombinant LCN2 pretreatment promotes acute lung inflammation in LPS-treated mice

**Figure 3:**
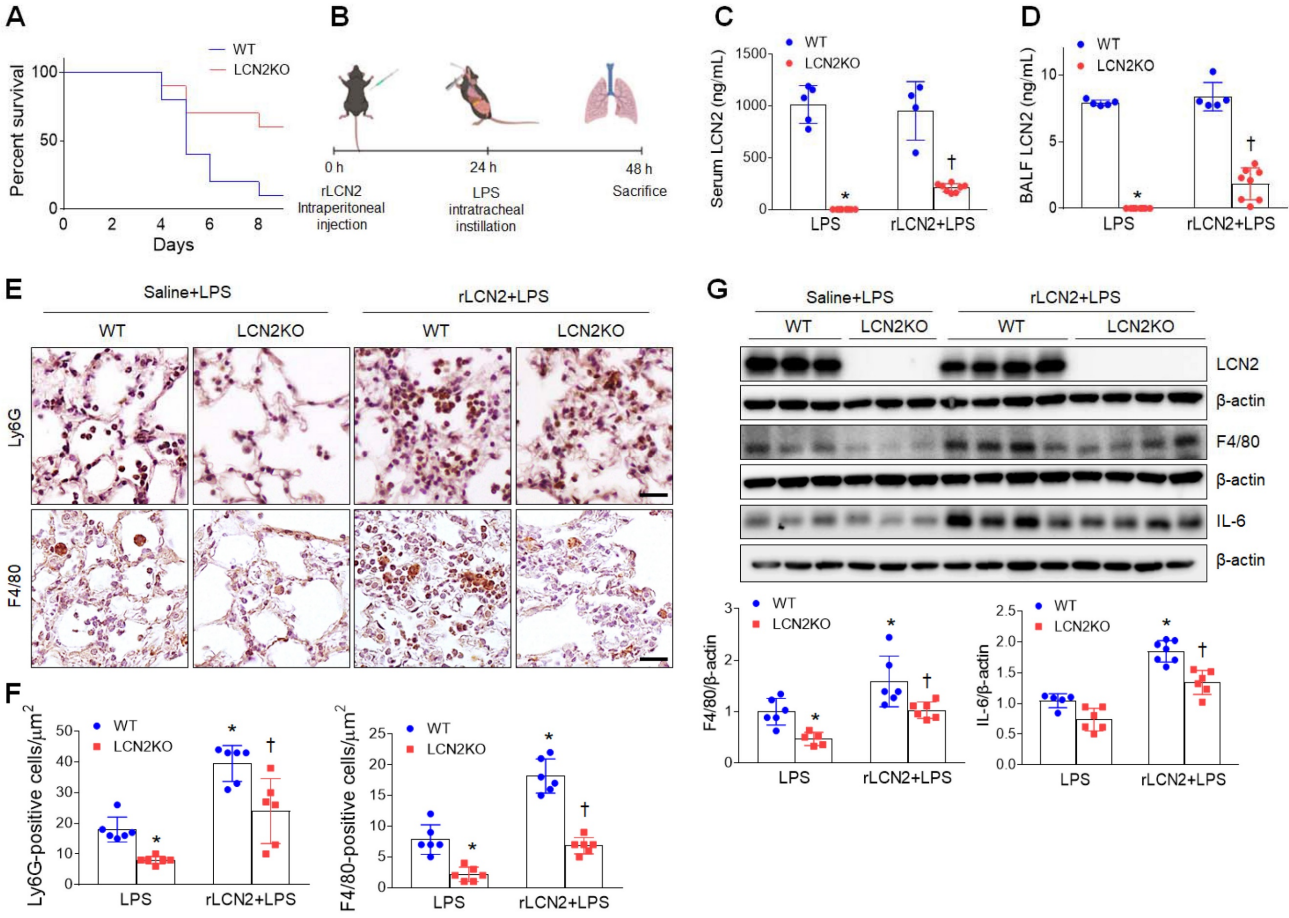
** Pretreatment with rLCN2 promotes acute lung inflammation in LPS-treated WT and LCN2 KO mice. (A)** Percent survival following intratracheal instillation with 20 mg/kg LPS after rLCN2 pretreatment. (n = 10). **(B)** Experimental schematic of rLCN2 pretreatment in LPS-treated mice. **(C-D)** ELISA analyses of LCN2 concentrations in serum (C) and BALF (D). (n = 4-8). **(E)** Representative images of Ly6G and F4/80 immunoreactivity in lung sections of rLCN2+LPS-treated WT and LCN2 KO mice. Scale bar, 20 μm. **(F)** Ly6G- and F4/80-positive cells were counted and analyzed in two fields (300x300 μm^2^) for each slide (n = 6). **(G)** Western blot and quantification of LCN2, F4/80, and IL-6 proteins in lung tissues (n = 5-7). β-actin served as a loading control. Differences between two groups were evaluated using unpaired Student's* t*-tests. *P < 0.05 versus LPS-treated WT. †*P* < 0.05 versus rLCN2+LPS-treated WT. All data are presented as mean ± SEM.

As expected, after rLCN2 pretreatment, LPS (20 mg/mL)-treated LCN2 KO mice exhibited a higher survival rate than LPS-treated WT mice (Figure 3A). To further test the hypothesis that LCN2 promotes acute lung inflammation, LPS (1 mg/mL)-treated mice were intraperitoneally pretreated with rLCN2 (Figure 3B). Although both serum and BALF LCN2 proteins were measured in LCN2 deficient mice, they were prominently reduced in rLCN2+LPS-treated LCN2 KO mice compared to rLCN2+LPS-treated WT mice (Figure 3C, D). Immunohistochemical study revealed that rLCN2 pretreatment increased Ly6G-positive neutrophils and F4/80-positive macrophages in lung sections of LPS-treated WT mice, but many Ly6G- and F4/80-positive cells in rLCN2+LPS-treated WT mice were significantly reduced by LCN2 deletion (Figure 3E, F). Furthermore, rLCN2 increased lung F4/80 and IL-6 protein levels in LPS-treated WT mice, whereas LCN2 deletion significantly attenuated theirs protein levels in rLCN2+LPS-treated mice (Figure 3G). These findings indicate that exogenous LCN2 can promote acute inflammation in LPS-induced acute lung injury.

### Recombinant LCN2 pretreatment promotes lung oxidative stress and iron overload in LPS-treated mice

**Figure 4:**
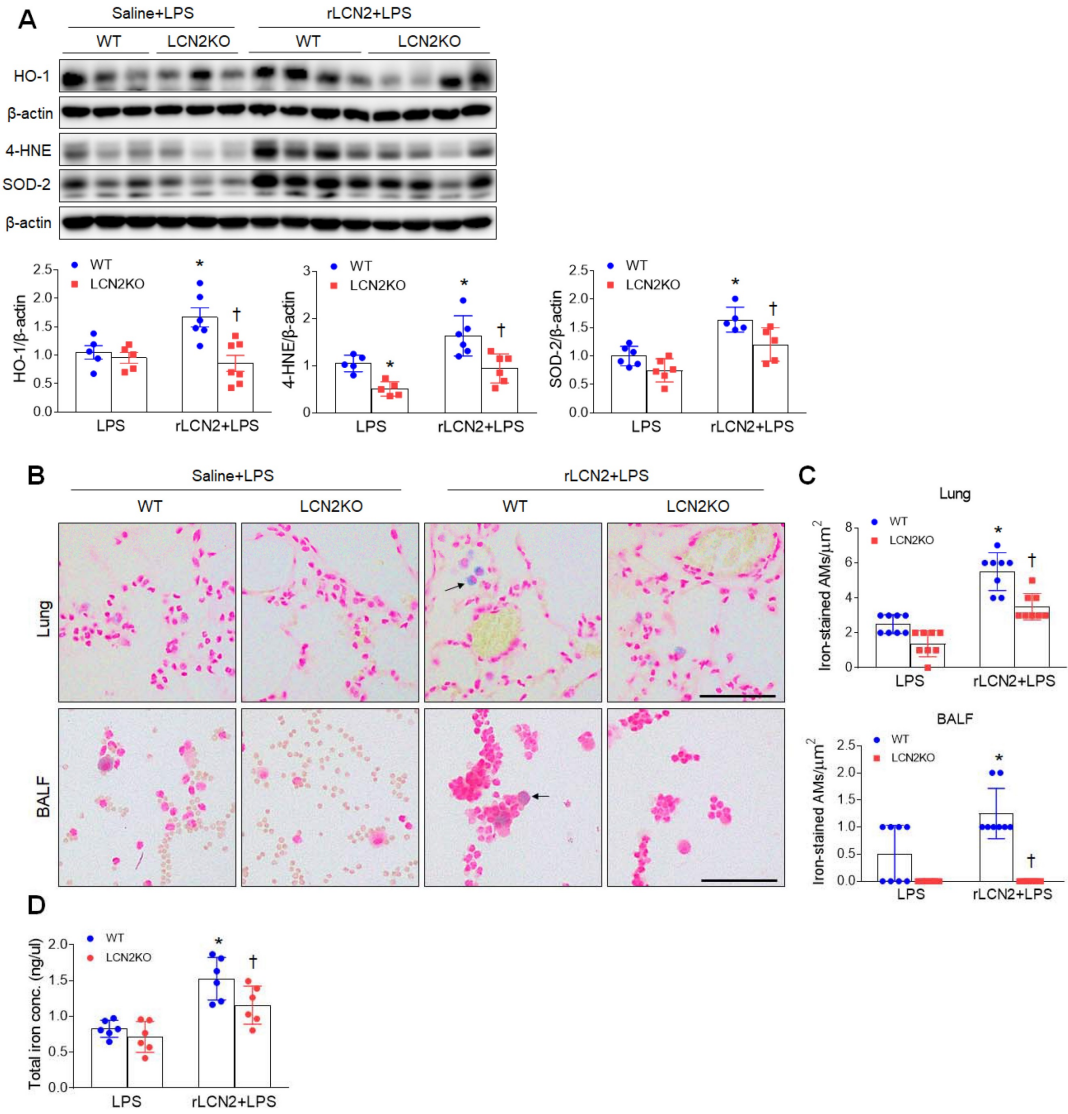
** Pretreatment with rLCN2 promotes lung oxidative stress and iron deposition in LPS-treated WT and LCN2 KO mice. (A)** Western blotting and quantification of lung HO-1, 4-HNE, and SOD-2 protein levels in lung tissues. β-actin served as a loading control. (n = 5-7). **(B)** Representative images of Perls Prussian blue iron staining in lung sections and BALF slides. Arrows indicate iron-stained alveolar macrophages. Scale bar, 50 μm. (n = 4 mice). **(C)** In lung sections and BALF slides (B), iron-positive alveolar macrophages were counted and analyzed in four fields (100x100 μm^2^) for each slide (n = 6). **(D)** Quantification of total lung iron levels using an iron assay kit. (n = 6 mice). Differences between two groups were evaluated using unpaired Student's* t*-tests. *P < 0.05 versus LPS-treated WT. †*P* < 0.05 versus rLCN2+LPS-treated WT. All data are presented as mean ± SEM.

Given that LCN2 increased iron uptake in alveolar macrophages, we evaluated whether exogenous LCN2 pretreatment affects oxidative stress in LPS-treated mice. Western blot analysis showed that rLCN2 increased lung HO-1, 4-HNE, and SOD-2 protein levels in LPS-treated WT mice, whereas LCN2 deletion significantly reduced these protein levels in rLCN2+LPS-treated mice (Figure 4A). Double immunofluorescence revealed that many co-localized HO-1 (or 4-HNE) and F/480 (or CD11b)-positive macrophages in rLCN2+LPS-treated WT mice were significantly reduced by LCN2 deletion (Supplementary Figure 6 and 7). Furthermore, rLCN2 increased the number of iron-stained AMs in lung sections and BALF of LPS-treated WT mice, whereas LCN2 deletion prominently attenuated the increased number of iron-stained macrophages in the lung section and BALF of rLCN2+LPS-treated mice (Figure 4B, C). Notably, we found that total lung iron levels were higher in rLCN2+LPS-treated WT mice than LPS-treated WT mice, and were lower in rLCN2+LPS-treated LCN2 KO mice compared to rLPS+LPS-treated WT mice (Figure 4D). Taken together, these results suggest that exogenous LCN2 may promote acute lung injury by iron accumulation-induced oxidative stress.

### Exogenous LCN2 increases proinflammatory cytokines in RAW264.7 cells

**Figure 5:**
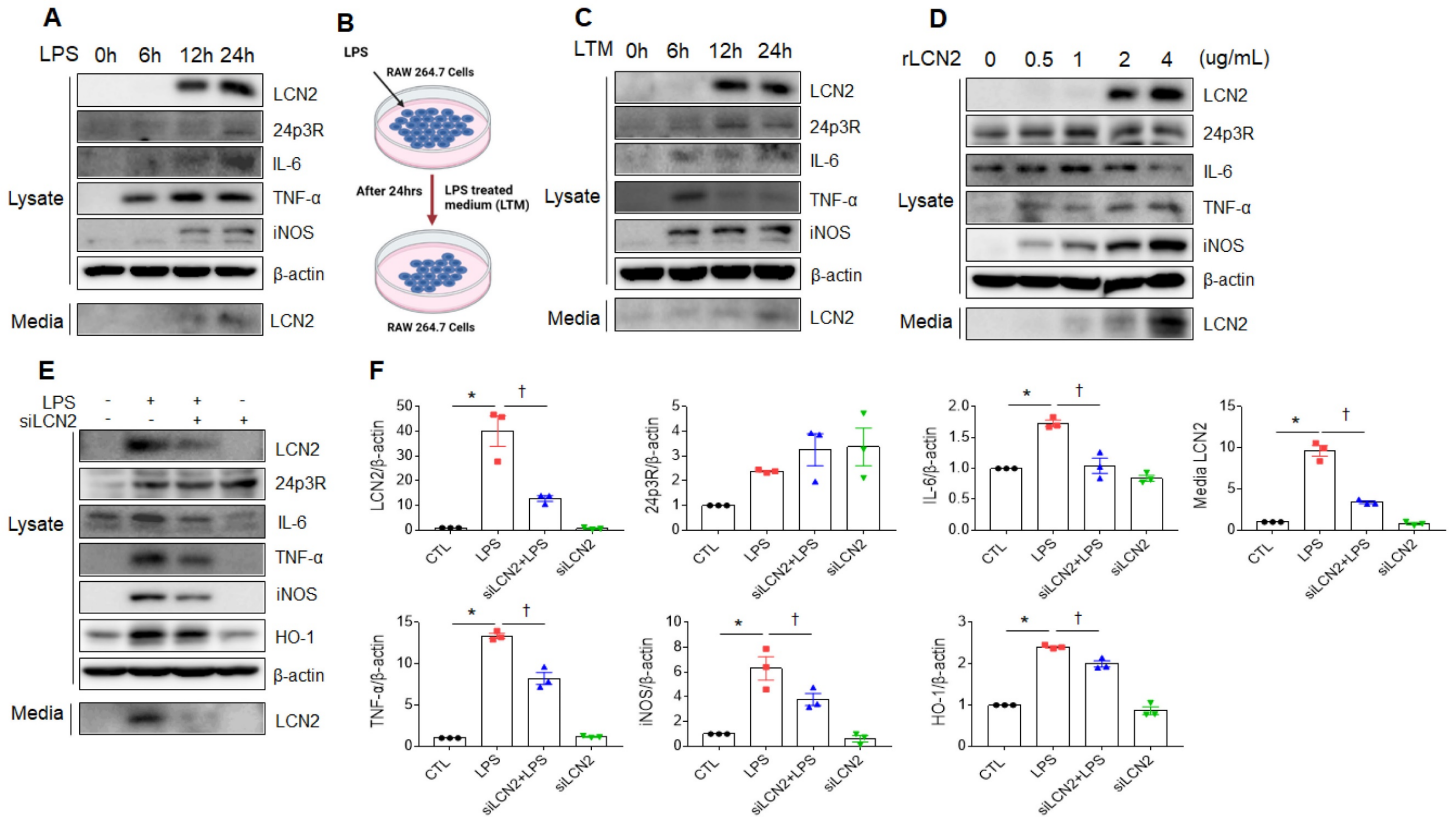
** Effect of LCN2 deletion on proinflammatory cytokines in LPS-treated RAW264.7 cells**. **(A)** Western blot analysis of cellular lysate LCN2, 24p3R, IL-6, TNF-α, iNOS, and media LCN2 in LPS-treated RAW264.7 cells. **(B)** Schematic drawing of LPS-treated RAW264.7 cells medium (LTM) treatment method in LTM-treated RAW264.7 cells.** (C)** Western blot analysis of cellular lysate LCN2, 24p3R, IL-6, TNF-α, iNOS, and media LCN2 in LTM-treated RAW264.7 cells. **(D)** Western blot analysis of cellular lysate LCN2, 24p3R, IL-6, TNF-α, iNOS, and media LCN2 in recombinant LCN2 (rLCN2)-treated RAW264.7 cells. **(E-F)** Western blot analysis (E) and quantification (F) of cellular lysate LCN2, 24p3R, IL-6, TNF-α, iNOS, HO-1, and media LCN2 in siLCN2+LPS-treated RAW264.7 cells from three independent experiment. β-actin served as a loading control. Differences between four groups were evaluated using two-way ANOVA followed by Tukey's multiple comparisons test. *P < 0.05 versus CTL. †*P* < 0.05 versus LPS. All data are presented as mean ± SEM.

We next verified whether LPS or exogenous LCN2 affects LCN2 expression in RAW264.7 cells. As shown in figure 5A, LCN2, 24p3R, IL-6, TNF-α, iNOS, and secreted LCN2 were increased in a time-dependent manner. Consistent with LPS treatment, these proteins were elevated in RAW264.7 cells following LPS-treated medium (LTM) treatment (Figure 5B, C). Furthermore, we also found that rLCN2 increases these proteins in a dose-dependent manner (Figure 5D). To determine whether LCN2 silencing inhibits proinflammatory cytokines in LPS-treated RAW264.7 cells, we used siLCN2. siLCN2 treatment significantly reduced LPS-induced LCN2, IL-6, TNF-α, iNOS, HO-1, and secreted LCN2 protein levels in LPS-treated RAW264.7 cells (Figure 5E, F). These results indicate that LCN2 promotes the secretion of proinflammatory cytokines in activated macrophages.

### Iron chelator inhibits LCN2-induced inflammation and oxidative stress in LTM or rLCN2-treated RAW264.7 cells

**Figure 6:**
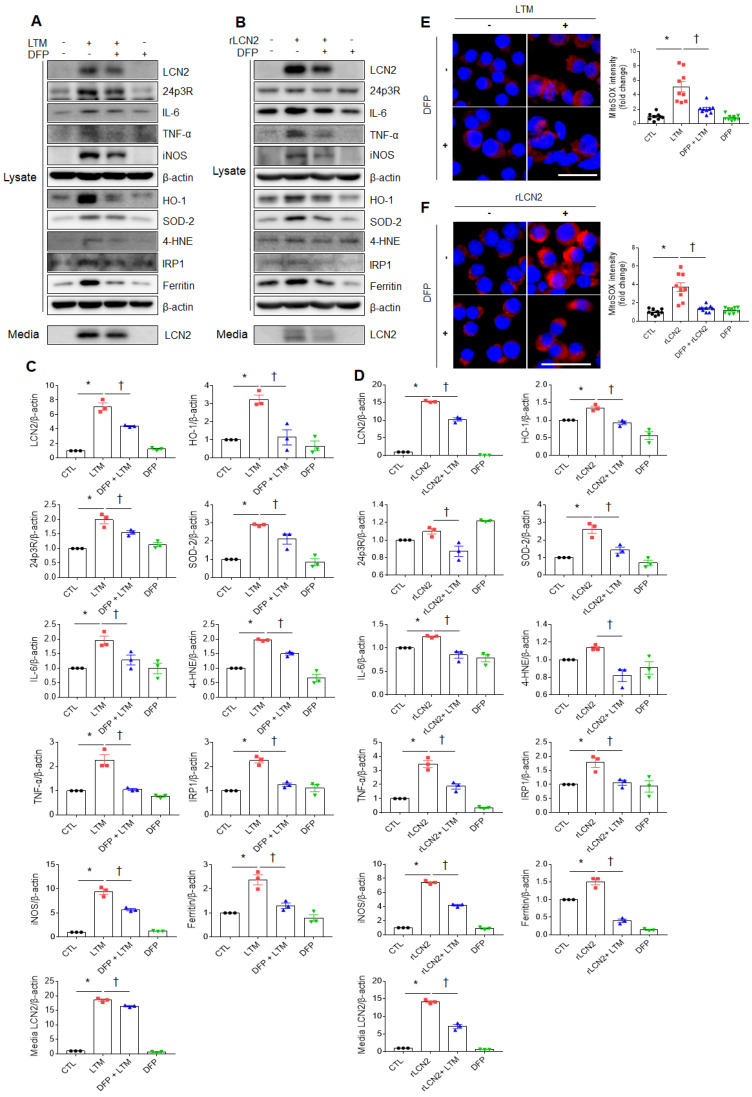
** Effects of iron chelator deferiprone on inflammation and oxidative stress in LTM or rLCN2-treated RAW264.7 cells. (A, C)** Western blot analysis and quantification of cellular lysate LCN2, 24p3R, IL-6, TNF-α, iNOS, HO-1, SOD-2, 4-HNE, IRP1, ferritin, and media LCN2 in deferiprone (DFP)+ LTM-treated RAW264.7 cells from three independent experiment. β-actin served as a loading control. **(B, D)** Western blot analysis and quantification of cellular lysate LCN2, 24p3R, IL-6, TNF-α, iNOS, HO-1, SOD-2, 4-HNE, IRP1, ferritin, and media LCN2 in DFP+rLCN2-treated RAW264.7 cells from three independent experiment. β-actin served as a loading control. **(E-F)** Detection and quantification of superoxide by MitoSOX™ Red in DFP+LTM (E)- and DFP/rLCN2 (F)-treated RAW264.7 cells. DAPI was used for nuclear staining. Scale bar, 25 μm. Bar graphs indicate MitoSOX™ Red fluorescence intensity. Differences between four groups were evaluated using two-way ANOVA followed by Tukey's multiple comparisons test. *P < 0.05 versus CTL. †*P* < 0.05 versus LTM or rLCN2. All data are presented as mean ± SEM.

To assess the protective role of iron chelator on LPS-induced acute lung injury, iron chelator DFP was intraperitoneally given to WT and LCN2 KO mice (Supplementary Figure 8A). BALF-stained analysis showed that many neutrophils and iron-stained alveolar macrophages in LPS-treated WT mice were prominently reduced by LCN2 deletion (Supplementary Figure 8B). In particular, the numbers of neutrophils and iron-stained macrophages were lower in the BALF of LPS+DFP-treated WT and LCN2 KO mice than LPS-treated WT mice. To further assess the role of iron chelator on inflammation and oxidative stress, we depleted cellular iron in RAW264.7 cells using iron chelator DFP. DFP significantly decreased LTM or rLCN2-induced LCN2, 24p3R, IL-6, TNF-α, iNOS, and medium LCN2 proteins in RAW264.7 cells (Figure 6A-D). As expected, LTM- or rLCN2 -induced HO-1, SOD-2, and 4-HNE protein levels were also prominently reduced by DFP. In particular, increased iron responsive element binding protein 1 (IRP1) and iron-storage protein ferritin levels were significantly reduced by DFP (Figure 6A-D). Furthermore, increased intensities of MitoSOX in LTM or rLCN2-treated RAW264.7 cells were prominently inhibited by DFP (Figure 6E, F). Thus, these results suggest that ion chelator inhibits inflammation and oxidative stress in activated macrophages under elevated LCN2 levels.

### Iron chelator inhibits phagocytosis in LTM or rLCN2-treated RAW264.7 cells

**Figure 7:**
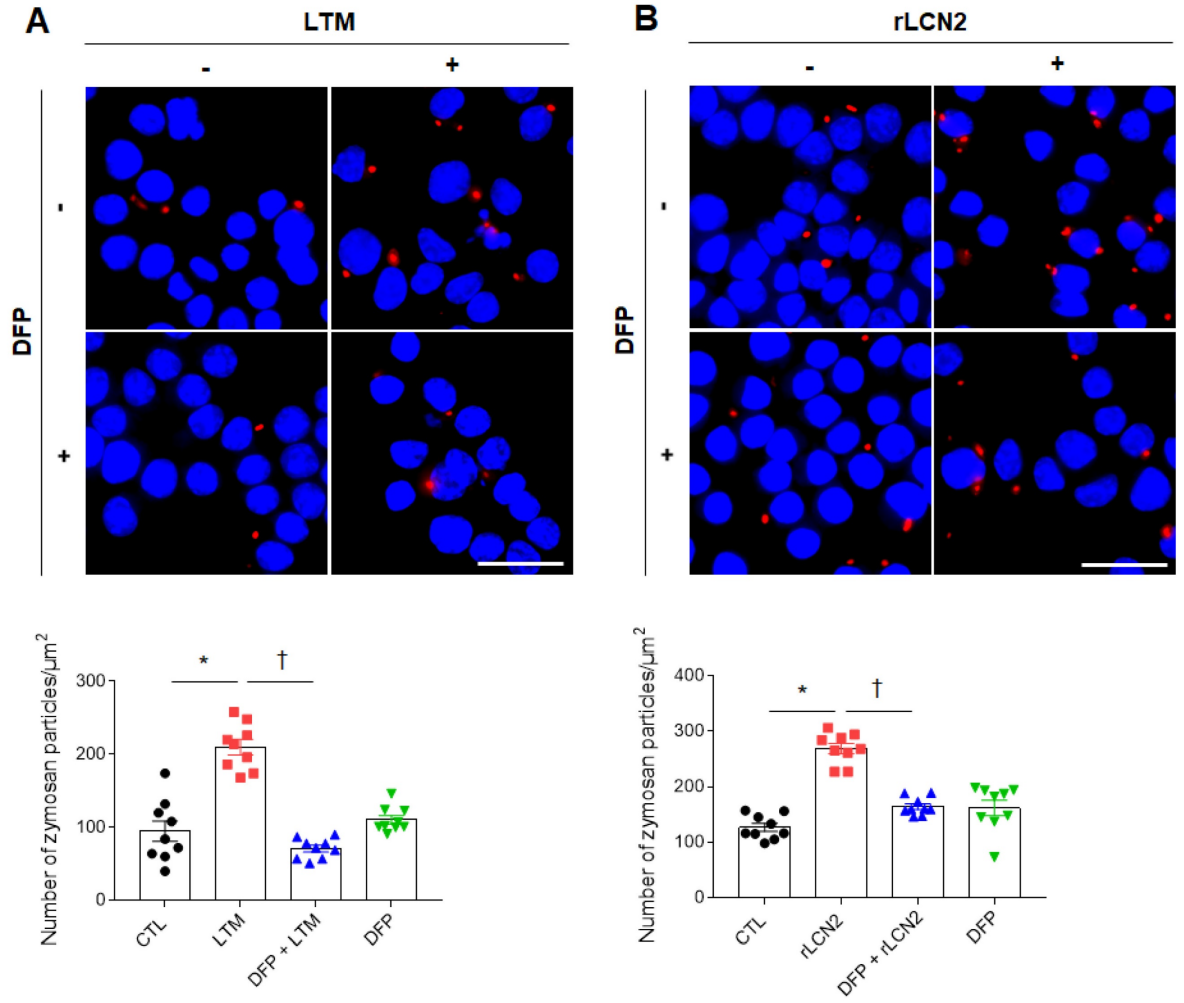
** Effects of iron chelator deferiprone on phagocytosis in LTM or rLCN2-treated RAW264.7 cells**. **(A-B)** Representative images obtained after 30 min incubation with zymosan red particle in deferiprone (DFP)+LTM (A)- and DFP+rLCN2 (B)-treated RAW264.7 cells. DAPI was used for nuclear staining. Scale bar, 25 μm. Bar graphs indicate the number of zymosan red particles. Differences between four groups were evaluated using two-way ANOVA followed by Tukey's multiple comparisons test. *P < 0.05 versus CTL. †*P* < 0.05 versus LTM or rLCN2. All data are presented as mean ± SEM.

Zymosan has been used as a model for the recognition of microbes by immune responses 26. After inflammatory stimuli in macrophages, phagocytic receptors on macrophages bind zymosan and stimulate particle engulfment 27. So, we measured phagocytosis using zymosan assay kit. As shown in supplementary figure 9, LCN2 knockdown significantly reduced the increased number of zymosan particles in LPS-treated RAW264.7 cells. To further determine the role of iron chelator on phagocytosis, we depleted cellular iron in LTM or rLCN2-treated RAW264.7 cells using DFP. DFP significantly reduced the increased number of zymosan particles in LTM or rLCN2-treated RAW264.7 cells (Figure 7). These findings provide evidence that iron chelator can inhibit LCN2-induced iron accumulation and may play an essential role in the regulation of inflammation and oxidative stress against acute lung injury.

## Discussion

In this study, we investigated that LCN2 is an inflammatory mediator that is elevated in LPS-treated mice and in patients with pneumonia. LCN2-deleted mice with a higher survival rate after LPS treatment had reduced lung inflammation and oxidative stress compared to LPS-treated WT mice. In contrast, rLCN2 pretreatment exacerbated acute lung inflammation and oxidative stress. In particular, we found that increased iron-stained alveolar macrophages can be associated with acute lung injury. Furthermore, DFP inhibited inflammation, oxidative stress, and phagocytosis in RAW264.7 cells with high LCN2 levels. Thus, our findings suggest that LCN2 may promote acute lung injury by enhancing macrophage iron accumulation.

The role of LCN2 in inflammatory conditions remains controversial, and experimental results vary depending on the selected agent (bacteria or LPS) and administration route (intraperitoneal or intratracheal). Classically, LCN2 bound to bacterial siderophores inhibits bacterial growth by preventing the utilization of host iron 12. It has been known that LCN2 is protective against intraperitoneal infection by *E. coli* or* Klebsiella pneumoniae* 12, 13*.* Intratracheal instillation of *E. coli* upregulates LCN2 in the lungs, and LCN2 loss results in increased mortality of these infected mice 28. Furthermore, LCN2 deficiency alters neutrophil homeostasis, impairing neutrophil migration and function in acute lung injury model using *E. coli* 29. In contrast, LCN2 impairs immune responses and bacterial clearance of *Streptococcus pneumonia* and increases mortality 30. Consistent with a recent study that LCN2 silencing inhibits lung inflammation in neonatal mice 18, we showed that after intratracheal injection of LPS treatment, LCN2 deficient mice had higher survival rates rather than LPS-treated WT mice. Additionally, rLCN2 pretreatment reduced mortality in LPS-treated LCN2 KO mice. Thus, our findings indicate that LCN2 may have a detrimental role in acute inflammatory conditions following intratracheal LPS administration.

It has been reported that LCN2 is increased in a variety of cell types, including bronchial epithelial cells, type II pneumocytes, neutrophils, and macrophages 31, 32. Consistent with an increased percentage of neutrophils in the BALF from pneumonia patients (Supplementary Table 1), LPS-treated mice had an increased number of neutrophils compared to macrophages (Supplementary Figure 1 and 3). Our findings may be due to the infiltration of neutrophils through blood-air barrier breakdown by LPS. Notably, we found that LCN2-positive cells are more prominently observed in alveolar macrophages than neutrophils in the BALF of pneumonia patients. Furthermore, as shown in figure 1C, LCN2 was remarkably increased in the mature macrophage isolated from the BALF of LPS-treated WT mice. Although pro-inflammatory cytokines and LCN2-positive macrophages were increased in BALF from patients with pneumonia, we saw no significant difference in LCN2 levels in BALF samples between pneumonia and controls. Maybe, we suggest that control subjects in this study consisted of patients with stable chronic lung disease, which may affect LCN2 levels. Thus, our data suggest that LCN2-mediated activated macrophages could play an important role in the regulation of acute lung inflammation.

By scavenging iron and storing it into ferritin, alveolar macrophages, which are resident lung macrophages, play a critical role in iron sequestration 33, 34. In fact, LCN2 transports free iron into macrophages, and these irons causes oxidative stress in infectious conditions 19, 20, 35. Consistent with evidence that iron overload-induced M1 polarization increases the secretion of proinflammatory cytokines 25, we also found that LPS-treated mice had MI polarization including BALF (IL-1ß and IL-6) and iron-stained macrophages. In particular, many iron-storing ferritin-positive macrophages were observed in LCN2-positive cells in the lungs of LPS-treated WT mice. However, these findings were reversed by LCN2 deletion. Furthermore, LCN2 KO mice had low levels of total iron concentrations compared to rLCN2+LPS-treated WT mice. Notably, DFP significantly the increased numbers of neutrophils and iron-stained alveolar macrophages in LPS-treated mice. The present study supports that exogenous LCN2 including LPS, LTM, and rLCN2 upregulates proinflammatory cytokines, but DFP inhibits these increased IL-6, TNF-α, iNOS proteins in exogenous-treated RAW264.7 cells. Taken together, our results indicate that LCN2-mediated iron sequestration may be associated with acute lung inflammation.

In addition to inhibiting siderophore-dependent bacterial iron uptake, LCN2 was found to have an impact on iron homeostasis and inflammatory response. However, it is still unclear to why siderophore-independent bacteria like *Streptococcus pneumoniae* cause macrophages to secrete LCN2. Warszawska et al. postulated that increased LCN2-induced IL-10 production could deactivate macrophages, prevent efficient bacterial clearance, and exacerbate the disease in a signal transducer and activator of transcription 3 (STAT3)-dependent pathway 30. So, we suggest that LCN2 may play an important critical role in macrophage polarization in different manners in response to different pathogens such as bacteria stains or diverse conditions.

Iron overload causes oxidative stress damage in the lung from coal dust and diesel particles containing iron-containing pollutants 36. Free radicals like nitric oxide and the superoxide anion are produced by these activated alveolar macrophages, which have exacerbated acute lung damage 37, 38. In this study, LPS-induced HO-1 and SOD-2 proteins in the mouse lungs were reduced by LCN2 deletion. There were increased numbers of HO-1 and 4-HNE-positive cells in pneumonia patients. Additionally, our data support that rLCN2-induced HO-1, SOD-2, and 4-HNE in LPS-treated WT mice are significantly reduced by LCN2 deficiency. Consistent with our results, 4-HNE-induced mitochondrial ROS exacerbates M1 polarization in obese mice 39, 40. LPS-induced activation of RAW264.7 cells also increased the 4-fold activity of IRP1, which has been known as a cytoplasmic RNA-binding protein that controls iron metabolism 41. They reported that NO acts as an intercellular stimulus to increase IRP1 activity in RAW264.7 cells. So we postulated that iron chelator could inhibit oxidative stress-related proteins and ferritin in exogenous LCN2-treated RAW264.7 cells. In accordance with previous study 38, DFP pretreatment resulted in reduced HO-1, SOD-2, 4-HNE, IRP1, ferritin, and MitoSOX and inhibited phagocytosis in RAW264.7 cells with high LCN2 level conditions.

On the other hand, alveolar macrophages have phagocytic functions for the removal of damaged cells, dust, and microorganisms. Macrophage phagocytosis function is essential for the resolution phage of acute lung injury 42. Apart from pathogens, the elimination of apoptotic neutrophils is significant to the resolution of acute lung injury. Using FACS analysis, we found that LPS treatment increases apoptotic cells in the BALF of LPS-treated WT mice compared to LPS-treated LCN2 KO mice. However, our findings are inconsistent with a recent study showing that LCN2 impairs phagocytic bacterial clearance of macrophages 29, 42. Although phagocytosis is beneficial during the acute phase of bacterial infection, excessive macrophage activation during acute lung injury can exacerbate lung damage. Here, our data show that after intratracheal LPS injection, LCN2 secreted by infiltrating neutrophils reciprocally activates macrophage-mediated inflammation and oxidative stress. Zymosan assay showed that increased zymosan particles in LTM or rLCN2-treated RAW264.7 cells are significantly attenuated by DFP. These findings further indicate that enhanced phagocytic function by acute inflammation could be regulated by iron chelator. Taken together, these results suggest that inhibiting LCN2-mediated iron uptake protects against acute lung inflammation by sequestrating iron or by inhibiting iron-mediated oxidative stress in activated macrophages.

There are several limitations of our study. First, the small sample sizes of patients and controls with stable chronic lung disease may have obscured significance differences of LCN2 in BALF. Second, we used LPS as the ALI insult, so the role of LCN2 during direct pathogen infection was not evaluated, which may limit the generalizability of our findings. Third, serum and BALF samples were obtained at patient admission, so evolution over time and with treatment was not evaluated. Fourth, we only investigated alveolar macrophages, although LCN2 also plays roles in neutrophils, another important innate immune cell subset that protects against infection.

In conclusion, we demonstrated that alveolar macrophages are responsible for inflammation, oxidative stress, and phagocytosis in acute lung injury via LCN2-mediated iron overload. Our findings showed that excessive iron accumulation within alveolar macrophages contributes to M1 polarization and causes oxidative stress. *In vitro* study supported that iron chelator inhibits inflammation, oxidative stress, and macrophage phagocytosis under elevated LCN2 condition levels. So, the present study strongly suggests that LCN2-targeting the polarization of macrophages has potential advantages and therapeutic targets for acute lung injury or pneumonia.

## Supplementary Material

Supplementary figures and tables.

## Abbreviations

LCN2: Lipocalin-2
LPS: lipopolysaccharide
KO: knockout
rLCN2: recombinant LCN2
BALF: bronchoalveolar lavage fluid
ARDS: acute respiratory distress syndrome
HO: heme oxygenase-1
SOD-2: superoxide dismutase-2
FACS: fluorescence-activated cell sorting
ELISA: enzyme-linked immunosorbent assay
DFP: deferiprone
MitoSOX: Mitochondrial superoxide
IL-6: interleukin-6
4-HNE: 4-Hydroxynonenal
iNOS: inducible nitric oxide synthase
IRP1: iron regulatory proteins 1
TNF-α: tumor necrosis factor-α
H&E: Haematoxylin & eosin
siLCN2: LCN2-specific small interfering RNA
LTM: medium containing LCN2
