# Supplementary material for: Lipocalin-2 promotes acute lung inflammation and oxidative stress by enhancing macrophage iron accumulation
Source: Int J Biol Sci. 2023 Feb 13;19(4):1163–77. doi: 10.7150/ijbs.79915 (PMC10008694; doi:10.7150/ijbs.79915)
Supplement: Supplementary file 1 — Supplementary figures and tables. [file ijbsv19p1163s1.pdf]

Supplementary Table 1. Comparison of characteristics between controls and patients with pneumonia

Variables		Pneumonia	Control	*P*-value
		n = 26	n = 10	
**Age, years**		70.5 ± 11.4	60.1 ± 17	0.071
**Sex, male**		23 (88.5)	8 (80)	0.603
**BMI, kg/m^2**		19.6 ± 3.6	22.2 ± 2.6	0.07
**Type 2 diabetes mellitus**		15 (57.7)	0 (0)	0.002
**Hypertension**		12 (46.2)	1 (10)	0.06
**CLD**		4 (15.4)	0 (0)	0.559
**CKD**		1 (3.8)	1 (10)	0.484
**COPD**		6 (23.1)	1 (10)	0.645
**ILD**		5 (19.2)	8 (80)	0.001
**Septic shock**		18 (69.2)	0 (0)	<0.001
**ARDS**		17 (65.4)	0 (0)	<0.001
**WBC, × 10^3/mm^3**		13.6 ± 9.7	8.5 ± 5.7	0.044
**Hb, g/dL**		10.5 ± 1.7	13.1 ± 2.5	0.005
**PLT, × 10^3/mm^3**		177.8 ± 130	285.7 ± 65.6	<0.001
**C-reactive protein, mg/dL**		20.1 ± 9.9	3.4 ± 4.1	<0.001
**Procalcitonin, ng/mL**		7.3 ± 16.1	0.1 ± 0.1	<0.001
**Serum LCN2,ug/ml**		1.2 ± 0.1	0.7 ± 0.1	0.008
**Oxygen therapy**	**No oxygen**	0	8 (80)	<0.001
	**Nasal cannula**	0	2 (20)	
	**High-flow nasal cannula**	5 (19.2)	0	
	**Invasive mechanical ventilation**	21 (80.8)	0	
**BALF cell analysis**	**Neutrophil, %**	90.1 ± 11.8	16.8 ± 21.1	<0.001
	**Lymphocyte, %**	5 ± 6.7	40 ± 34.1	<0.001
	**Eosinophil, %**	0.1 ± 0.4	5.7 ± 11.6	<0.001
	**Macrophage, %**	4.8 ± 6.1	37.5 ± 33.9	<0.001
	**LCN2, ng/ml**	37.4 ± 1.8	38.6 ± 1.2	0.177

ARDS, acute respiratory distress syndrome; BALF, bronchoalveolar lavage fluid; CKD, chronic kidney disease; CLD, chronic lung disease; COPD, chronic obstructive pulmonary disease; Hb, hemoglobin; ILD, interstitial lung disease; PLT, platelet

Supplementary Table 2. List of primary antibodies

Antibody	Company	Catalog No.	Dilution(s)	Applications	Source
4-HNE	Abcam	ab46545	1:1000	WB	Rabbit
4-HNE	Abcam	ab48506	1:200	IF	Mouse
24p3R	Millipore	ABC846	1:1000	WB	Rabbit
CD11b	Abcam	ab52478	1:200	IF	Rabbit
F4/80	Santa Cruz	sc-377009	1:1000, 1:200, 1:200	WB, IHC, IF	Mouse
Ferritin	Abcam	ab75973	1:2000, 1:200	WB, IF	Rabbit
HO-1	Enzo Life	SPA-895	1:1000, 1:200	WB, IF	Rabbit
iNOS	DB Biotech	DB-003	1:1000	WB	Rabbit
IRP1	Santa Cruz	sc-166022	1:500	WB	Mouse
IL-6	MyBio Science	mbs3007753	1:1000	WB	Rabbit
IL-6	Santa Cruz	sc-57315	1:200	IF	Mouse
LCN2	R&D	AF1857	1:1000, 1:200, 1:200	WB, IHC, IF	Goat
Ly6G	Abcam	ab25377	1:200, 1:200	IHC, IF	Rat
SOD-2	Santa Cruz	sc-133134	1:1000	WB	Mouse
TNF-α	Santa Cruz	Sc-1351	1:1000	WB	Goat
β-actin	Sigma	A5441	1:3000	WB	Mouse

IF, immunofluorescence; IHC, immunohistochemistry; WB, western blot; 4-HNE, 4-Hydroxynonenal; HO-1, heme oxygenase-1; IL-6, interleukin-6; iNOS, inducible nitric oxide synthase; IRP1, iron regulatory protein 1; LCN2, lipocalin-2; SOD-2, superoxide dismutase-2; TNF-α, tumor necrosis factor-α

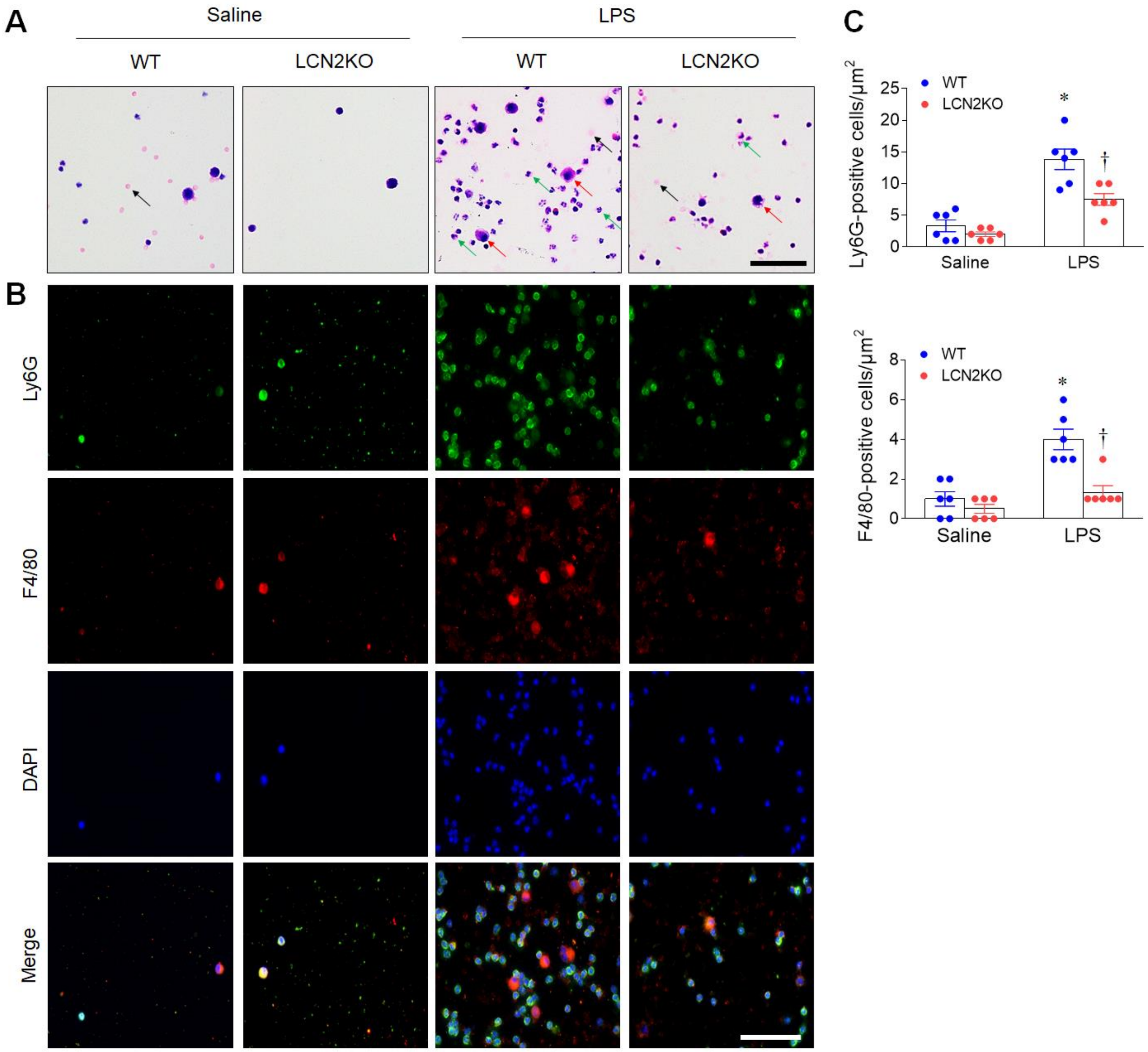

Supplementary Figure 1. Effects of LCN2 deletion on inflammation in the BALF of LPS-treated mice. (A) Representative images of Diff-Quik staining. Black, green, and red arrows indicate red blood cells, neutrophils, and macrophages, respectively. Scale bar, 50 μm. **(B)** Representative double immunofluorescence images of Ly6G-positive neutrophils and F4/80-positive alveolar macrophages. Nuclei were stained with DAPI. Scale bar, 50 μm. **(C)** Ly6G- and F4/80-positive cells were counted and analyzed in three fields (100x100 μm^2) for each slide (n = 6). Differences between the four groups

were evaluated using two-way ANOVA followed by Tukey's multiple comparisons test. *$P < 0.05$ versus WT saline. †$P < 0.05$ versus WT LPS. Data are presented as mean ± SEM.

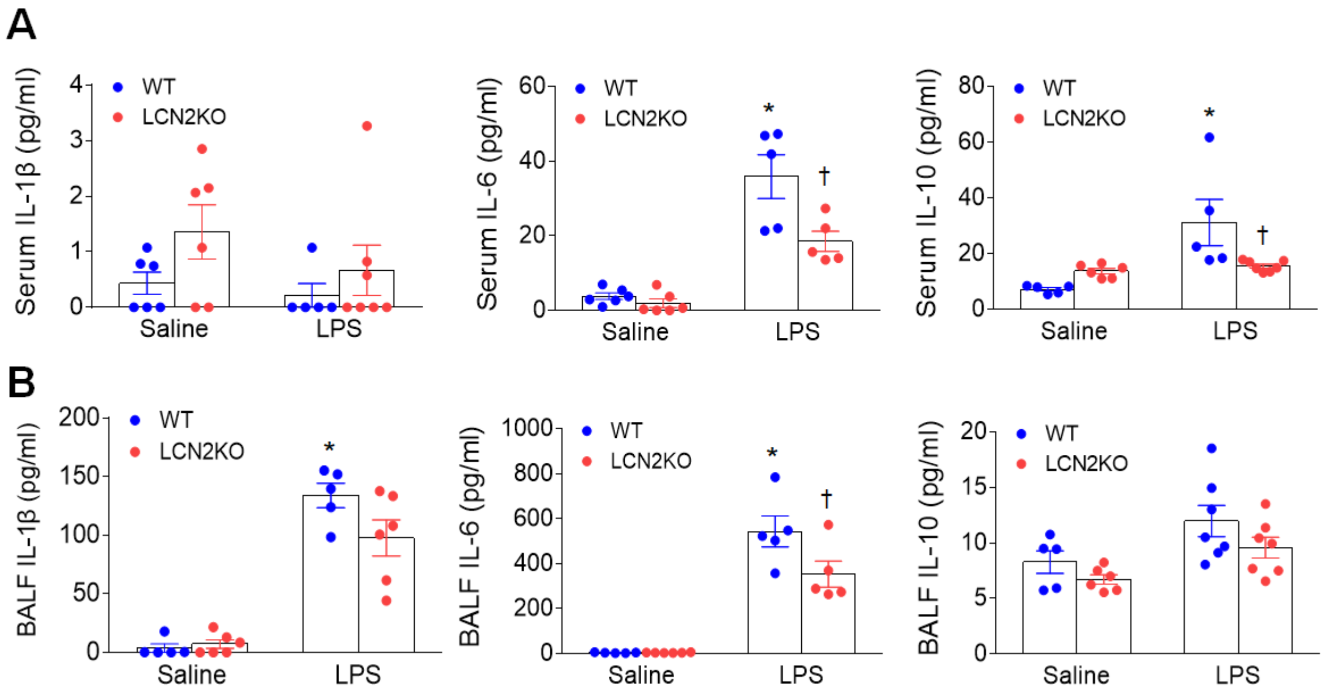

Supplementary Figure 2. Effects of LCN2 deletion on pro- and anti-inflammatory cytokines in serum and BALF of LPS-treated mice. (A-B) ELISA analysis of mouse IL-1β, IL-6, and IL-10 levels in serum (A) and BALF (B) (n = 5–7). Differences between the four groups were evaluated using two-way ANOVA followed by Tukey's multiple comparisons test. *$P < 0.05$ versus WT saline. †$P < 0.05$ versus WT LPS. Data are presented as mean ± SEM.

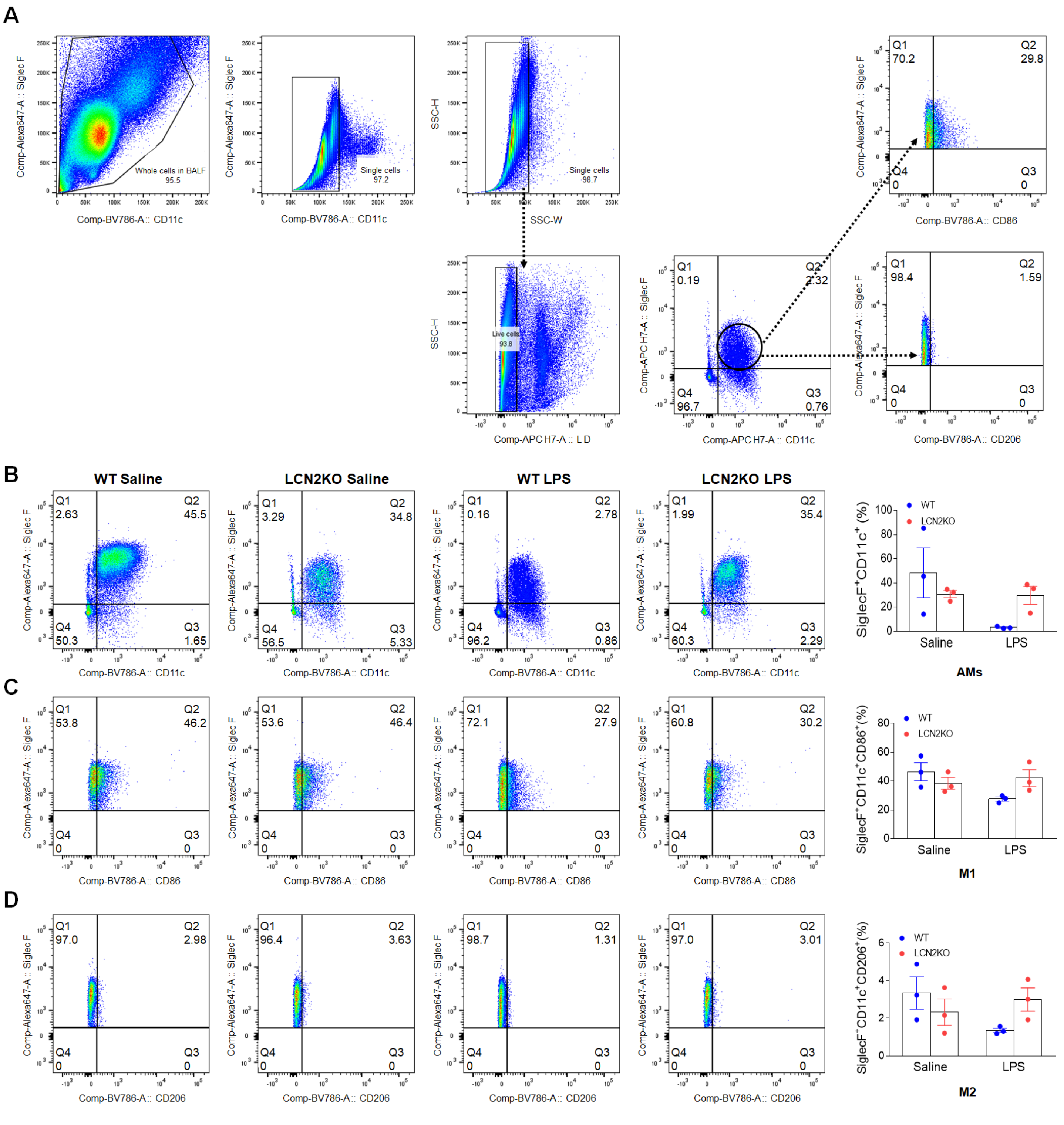

Supplementary Figure 3. Effects of LCN2 deletion on macrophage polarization in the BALF of LPS-treated mice. (A) Gating strategy of flow cytometry analysis to identify the M1 and M2 phenotypes of tissue-resident alveolar macrophages (AMs) in BALF. **B** Percentages of alveolar

macrophages in the BALF. **(C-D)** Percentages of M1 macrophages (C) and M2 macrophages (D) in AM in the BALF. Differences between the four groups (n = 3) were evaluated using two-way ANOVA followed by Tukey's multiple comparisons test. Data are presented as mean ± SEM.

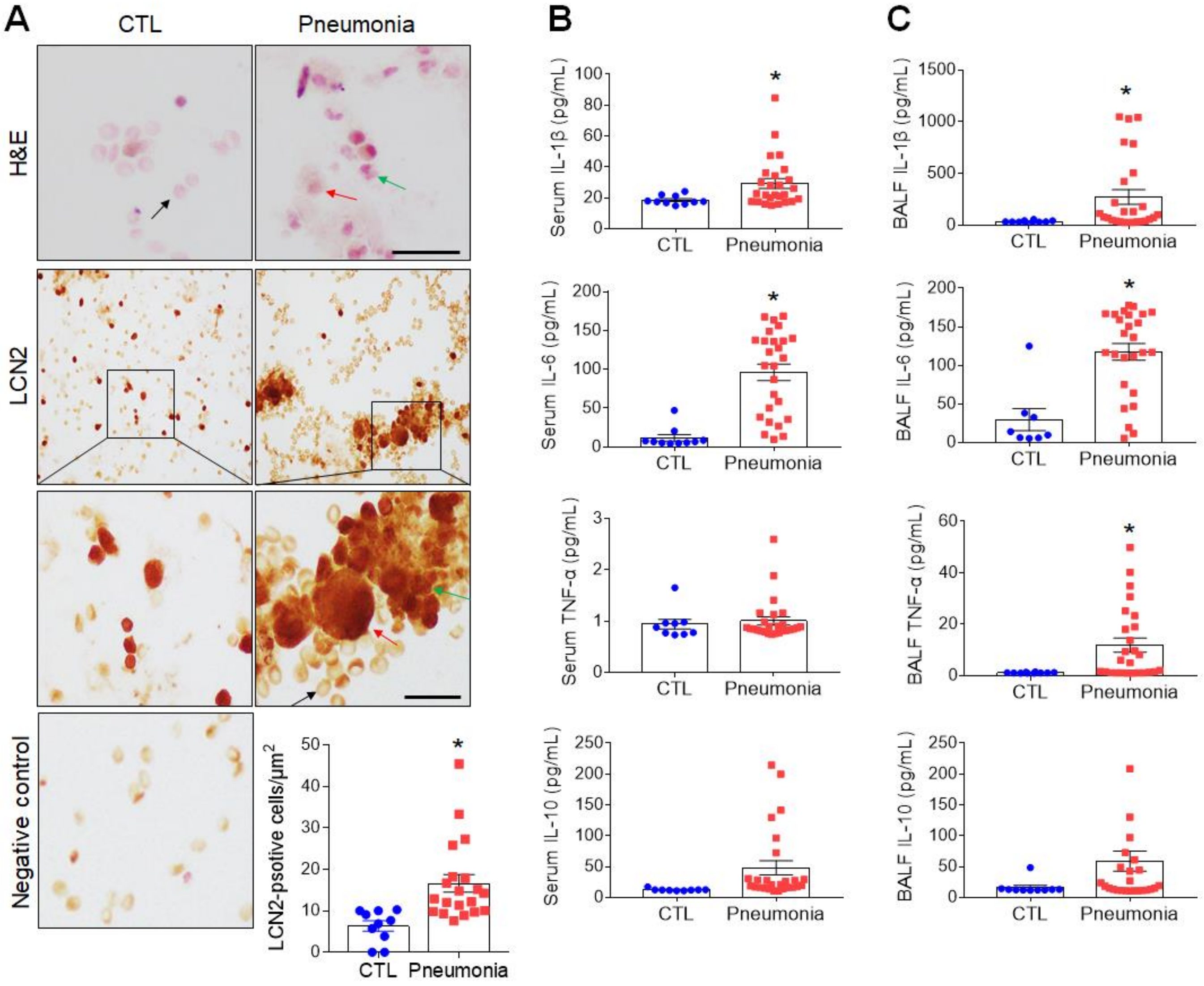

Supplementary Figure 4. Increased LCN2 expression in the BALF of patients with pneumonia. **(A)** Representative images of H&E and immunostaining of LCN2 in BALF slides from control subjects (CTL) and pneumonia patients. Scale bars, 50 μm (top) and 25 μm (bottom). The number of LCN2-positive cells with a round nucleus and large cytoplasm were counted and analyzed in two fields

(300x300 μm^2) for each slide. (n = 5-10). **(B-C)** ELISA analyses of IL-1β, IL-6, TNF-α, and IL-10 levels in serum (B) and BALF (C) from CTL (n = 10) and pneumonia patients (n = 26). Differences between the two groups were evaluated using unpaired Student's *t*-tests. *P < 0.05 versus CTL. Data are presented as mean ± SEM.

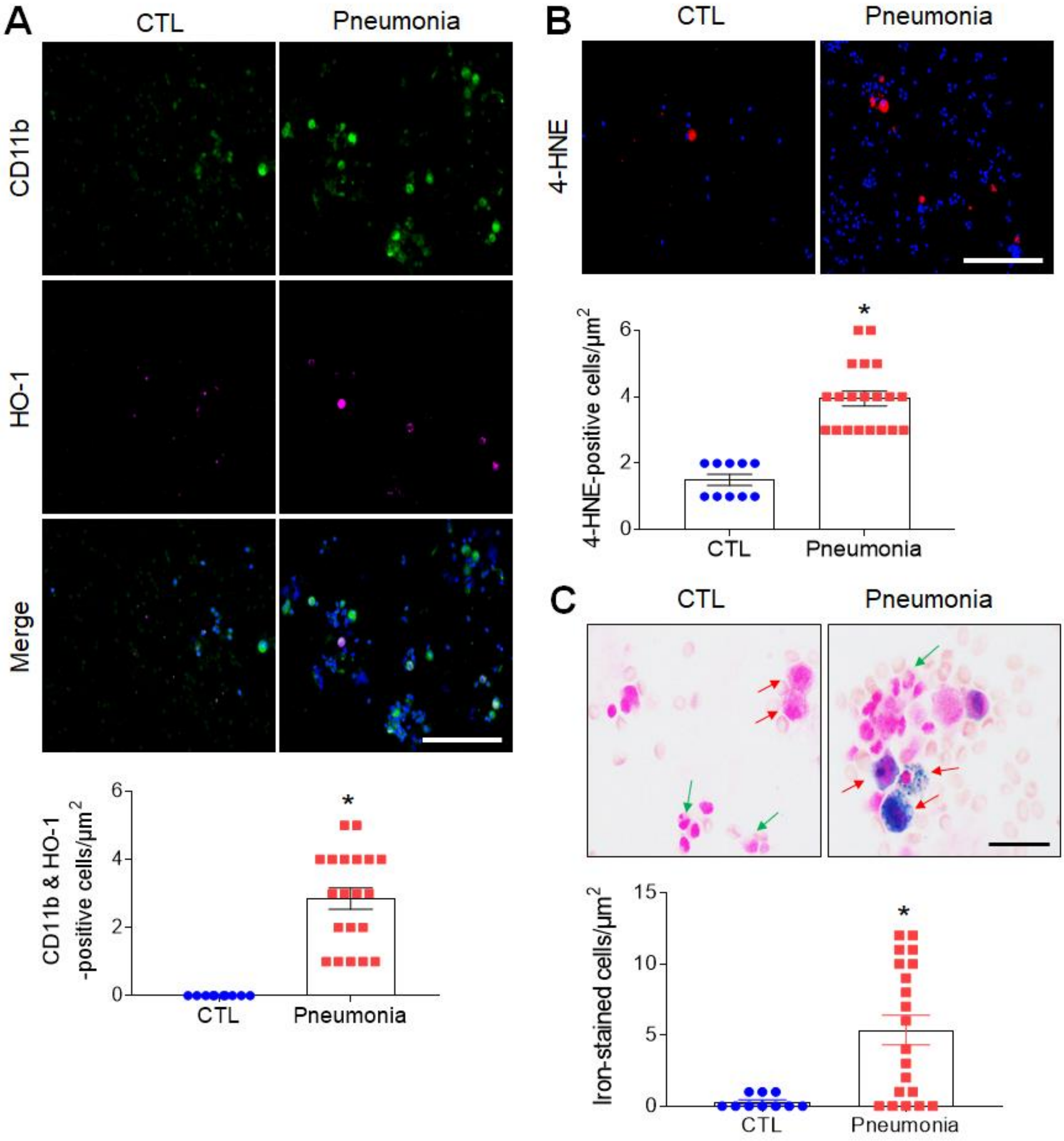

Supplementary Figure 5. Increased oxidative stress and iron accumulation in the BALF of patients with pneumonia. (A) Representative double immunofluorescence images of CD11b and HO-1 in the BALF slides of control subjects (CTL) and pneumonia patients. Nuclei were stained with DAPI. Scale bar, 100 μm. Co-localized CD11b and HO-1-positive cells were counted and analyzed in two fields (300x300 μm^2) for each slide. **(B)** Representative immunofluorescence images of 4-HNE in the BALF slides. Nuclei were stained with DAPI. Scale bar, 100 μm. 4-HNE-positive cells were counted and analyzed in two fields (300x300 μm^2) for each slide. **(C)** Representative images of Perls Prussian blue staining in BALF slides of CTL and pneumonia patients. Green and red arrows indicate neutrophils and macrophages, respectively. Scale bar, 25 μm. Iron-positive cells were counted and analyzed in two fields (100x100 μm^2) for each slide. Differences between the two groups (n = 5-10) were evaluated using unpaired Student's *t*-tests. *$P < 0.05$ versus CTL. Data are presented as mean ± SEM.

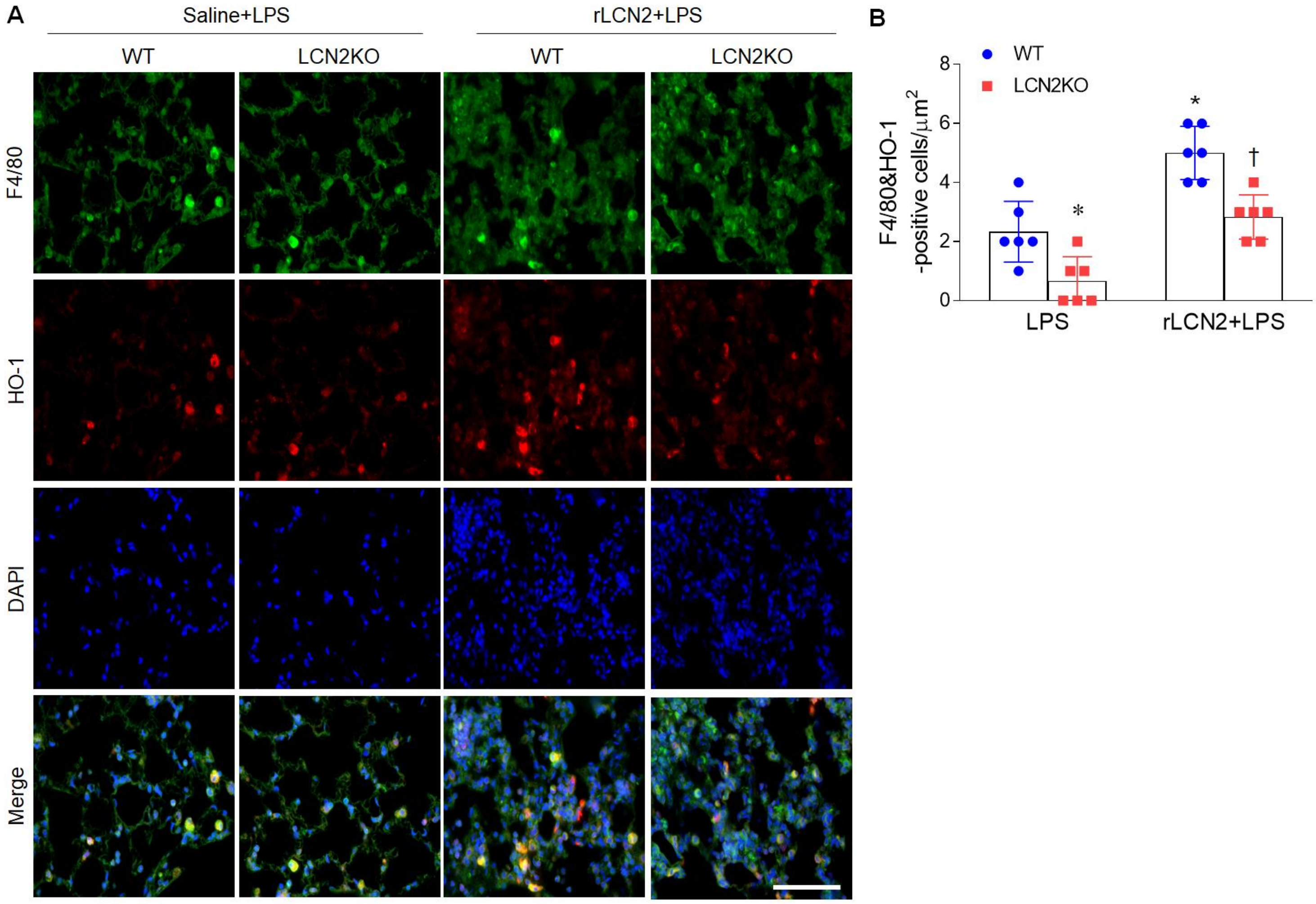

Supplementary Figure 6. Effects of rLCN2 pretreatment on HO-1 expression in LPS-treated WT and LCN2KO mice. (A) Representative double immunofluorescence images of F4/80 and HO-1 in lung tissues. **(B)** Bar graph shows the number of co-localized F4/80 and HO-1-positive cells. Co-localized positive cells were counted and analyzed in three fields (100x100 μm^2) for each slide. Nuclei were stained with DAPI. Scale bar, 50 μm. Differences between two groups (n = 6) were evaluated using unpaired Student's *t*-tests. *$P < 0.05$ versus Saline+LPS-treated WT. †$P < 0.05$ versus rLCN2+LPS-treated WT. All data are presented as mean ± SEM.

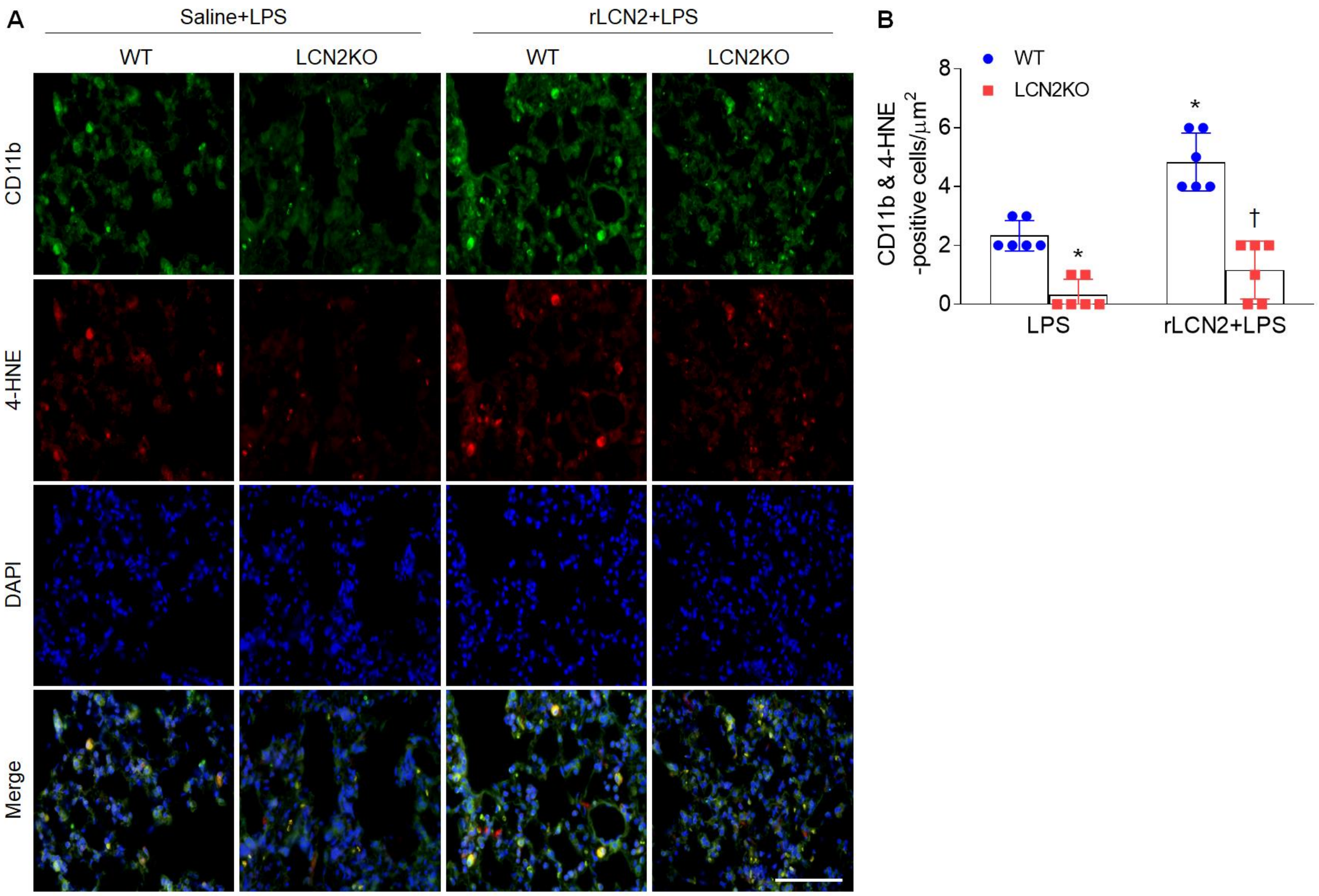

Supplementary Figure 7. Effects of rLCN2 pretreatment on 4-HNE expression in LPS-treated WT and LCN2KO mice. (A) Representative double immunofluorescence images of CD11b and 4-HNE in lung tissues. **(B)** Bar graph displays the number of co-localized CD11b and 4-HNE-positive cells. Co-localized positive cells were counted and analyzed in three fields (100x100 μm^2) for each slide. Nuclei were stained with DAPI. Scale bar, 50 μm. Differences between two groups (n = 6) were evaluated using unpaired Student's *t*-tests. **P* < 0.05 versus Saline+LPS-treated WT. †*P* < 0.05 versus rLCN2+LPS-treated WT. All data are presented as mean ± SEM.

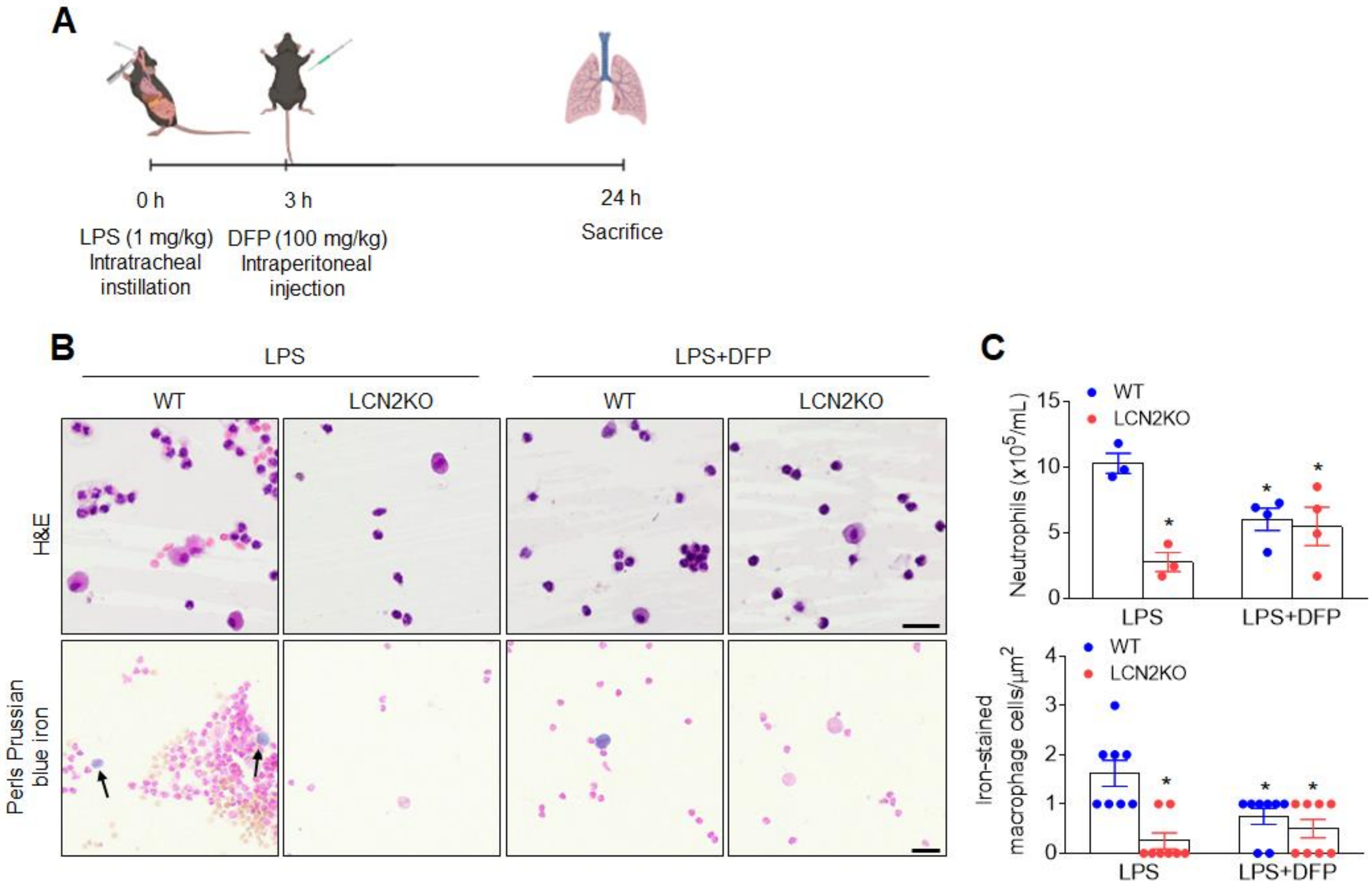

Supplementary Figure 8. Iron chelator inhibits acute lung injury in LPS-treated WT and LCN2 KO mice. (A) Experimental schematic of DFP treatment in LPS-treated WT and LCN2 KO mice. **(B)** Representative images of H&E and Perls Prussian blue staining in BALF slides. Arrows indicate iron-stained alveolar macrophages. Scale bar, 20 μm. **(C)** In BALF slides (B), H&E-stained neutrophils and iron-stained macrophages were counted and analyzed (n = 3-4). Differences between four groups were evaluated using two-way ANOVA followed by Tukey's multiple comparisons test. **P* < 0.05 versus LPS-treated WT. All data are presented as mean ± SEM.

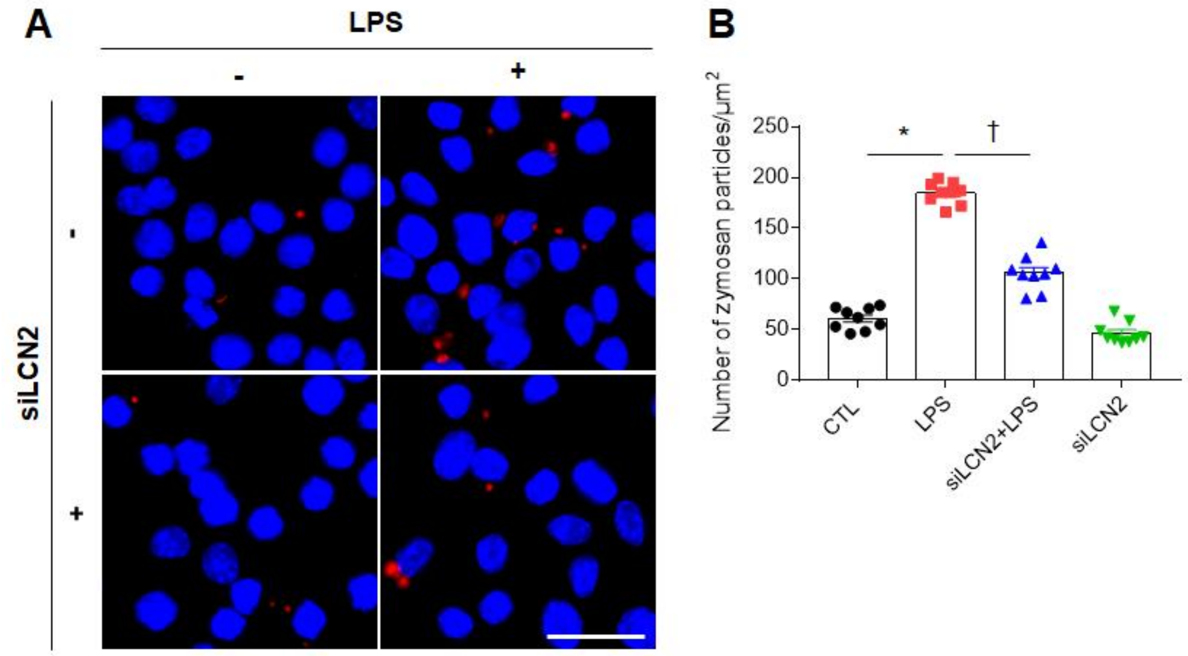

Supplementary Figure 9. Effects of LCN2 knockdown on phagocytosis in LPS-treated RAW264.7 cells. **(A)** Representative images obtained after 30 min incubation with zymosan red particle in siLCN2+LPS-treated RAW264.7 cells. DAPI was used for nuclear staining. Scale bar, 25 µm. **(B)** Bar graphs indicate the number of zymosan red particles. Differences between four groups were evaluated using two-way ANOVA followed by Tukey's multiple comparisons test. *$P < 0.05$ versus CTL. †$P < 0.05$ versus LPS. All data are presented as mean ± SEM.
